# Transcription of *Leishmania major* U2 small nuclear RNA gene is directed by extragenic sequences located within a tRNA-like and a tRNA-Ala gene

**DOI:** 10.1186/s13071-016-1682-3

**Published:** 2016-07-19

**Authors:** Saúl Rojas-Sánchez, Elisa Figueroa-Angulo, Rodrigo Moreno-Campos, Luis E. Florencio-Martínez, Rebeca G. Manning-Cela, Santiago Martínez-Calvillo

**Affiliations:** Unidad de Biomedicina, Facultad de Estudios Superiores Iztacala, Universidad Nacional Autónoma de México, Av. de los Barrios 1, Col. Los Reyes Iztacala, Tlalnepantla, Edo. de México CP 54090 Mexico; Departamento de Biomedicina Molecular, Centro de Investigación y de Estudios Avanzados del IPN, Av. IPN 2508, México, DF CP 07360 Mexico

**Keywords:** *Leishmania*, U2 snRNA, Promoter region, tRNA-like, Pseudouridine

## Abstract

**Background:**

*Leishmania* and other trypanosomatid parasites possess atypical mechanisms of gene expression, including the maturation of mRNAs by trans-splicing and the involvement of RNA Polymerase III in transcription of all snRNA molecules. Since snRNAs are essential for trans-splicing, we are interested in the study of the sequences that direct their expression. Here we report the characterization of *L. major* U2 snRNA promoter region.

**Results:**

All species of *Leishmania* possess a single U2 snRNA gene that contains a divergently-oriented tRNA-Ala gene in the upstream region. Between these two genes we found a tRNA-like sequence that possesses conserved boxes A and B. Primer extension and RT-qPCR analyses with RNA from transiently-transfected cells showed that transcription of *L. major* U2 snRNA is almost abolished when boxes A and B from the tRNA-like are deleted or mutated. The levels of the U2 snRNA were also highly affected when base substitutions were introduced into box B from the tRNA-Ala gene and the first nucleotides of the U2 snRNA gene itself. We also demonstrate that the tRNA-like is transcribed, generating a main transcript of around 109 bases. As pseudouridines in snRNAs are required for splicing in other organisms, we searched for this modified nucleotide in the *L. major* U2 snRNA. Our results show the presence of six pseudouridines in the U2 snRNA, including one in the Sm site that has not been reported in other organisms.

**Conclusions:**

Four different regions control the transcription of the U2 snRNA gene in *L. major*: boxes A and B from the neighbor tRNA-like, box B from the upstream tRNA-Ala gene and the first nucleotides of the U2 snRNA. Thus, the promoter region of *L. major* U2 snRNA is different from any other promoter reported for snRNAs. Pseudouridines could play important roles in *L. major* U2 snRNA, since they were found in functionally important regions, including the branch point recognition region and the Sm binding site.

**Electronic supplementary material:**

The online version of this article (doi:10.1186/s13071-016-1682-3) contains supplementary material, which is available to authorized users.

## Background

The information in eukaryotic protein-coding genes is frequently interrupted by introns, which have to be removed from the precursors of the mRNAs before they can be translated. The process in which introns are removed and exons are joined together is called splicing. This procedure is carried out in a stepwise coordinated fashion by a large ribonucleoprotein complex, named spliceosome, that consists of around 200 proteins [1] and five major small nuclear RNAs (snRNAs): U1, U2, U4, U5 and U6 [2, 3]. Each snRNA binds several specific proteins, as well as common proteins, to form a small nuclear ribonucleoprotein particle (snRNP). Among these proteins, the Sm proteins form a donut-shaped heptamer that interacts with a single-stranded region, the Sm site, within the U1, U2, U4 and U5 snRNAs [4]. On the other hand, the U6 snRNA binds Sm-like proteins that recognize a U-tract sequence located at the 3' end of the RNA [5].

The sequence and secondary structure of snRNAs are highly conserved, even between phylogenetically distant organisms [6]. All snRNAs are extensively post-transcriptionally modified and pseudouridine is the most abundant modified nucleotide in these RNA molecules [7]. Notably, most pseudouridines are located in functionally important regions of the snRNAs. In fact, several studies have demonstrated that pseudouridines play important roles in both snRNP assembly and splicing [8, 9].

Two different RNA polymerases (RNAP) are involved in the synthesis of snRNAs: U1 to U5 are transcribed by RNAP II, whereas U6 is transcribed by RNAP III [10]. In humans, the snRNA core promoter recognized by both RNAP II and III is composed of two elements: a proximal sequence element that is located between nucleotides −50 to −65, and a distal sequence element lying between positions −200 to −220, in relation to the transcription start site (TSS). Additionally, the RNAP III promoter contains a TATA box element situated around position −25 [11, 12].

Although splicing is widely conserved among eukaryotes, some organisms possess special forms of this mechanism to process mRNAs. For instance, protein-coding genes in *Leishmania* and other protozoan parasites that belong to the family Trypanosomatidae are organized into polycistronic gene clusters that are transcribed by RNAP II to generate polycistronic transcripts; and mature mRNAs are produced from these precursors by trans-splicing and polyadenylation. Trans-splicing is a process that adjoins a capped 39-nucleotide miniexon or spliced leader to the 5' termini of all the mRNAs [13]. Like cis-splicing, trans-splicing occurs via two transesterification reactions and requires the participation of snRNAs, but it involves the formation of a Y structure instead of a lariat intermediate [14, 15]. Although every snRNA has an essential participation in splicing, it has been shown that U2 and U6 snRNAs are key role players in the process, as they base-pair with each other to form the splicing catalytic core [16].

Another peculiarity of gene expression in trypanosomatids is that all snRNAs are transcribed by RNAP III [17]. Moreover, snRNA genes in *Trypanosoma brucei* have a divergently oriented tRNA gene, or a tRNA-like sequence, in their 5'-flanking region, and boxes A and B from the neighboring tRNA gene are essential for expression of the snRNAs [17–19]. Notably, the distance between box A from the tRNA gene and the snRNA gene is conserved, as it is usually ~104 bp in length [20, 21]. A third element located within the first 21 nucleotides of the snRNA coding region is also required for proper transcription initiation [17, 22]. However, not every snRNA gene needs all three sequence elements for transcription, as box B is dispensable for the *in vitro* synthesis of the U6 snRNA [22]. Also, *in vivo* expression of the U1 snRNA does not require an element located within the snRNA itself [19]. Moreover, box A of the associated tRNA gene is dispensable for U4 snRNA transcription in the related trypanosomatid *Leptomonas collosoma* [23].

Little attention has been paid to the study of snRNAs in *Leishmania*. In the present study we have analyzed the sequences that direct transcription of the U2 snRNA gene in *L. major*, the causative agent of cutaneous leishmaniasis in the Old World. As reported in other trypanosomatids, analysis of the genomic context of the U2 snRNA gene allowed us to identify a tRNA-like sequence located in the 5' flanking region that contains typical boxes A and B. By performing RT-qPCR analysis we found that these two elements, together with an intragenic sequence, are required for transcription of the U2 snRNA. Unlike other snRNAs in trypanosomatids, our results showed that an additional box B that resides on a tRNA-Ala gene located 211 bp upstream the U2 snRNA gene, is also important for the optimal transcription of this gene in *L. major*.

## Methods

### *Leishmania major* culture and transfection

Promastigotes from *L. major* MHOM/IL/81/Friedlin (LSB-132.1) were grown in BM medium (1× M199 medium pH 7.2, containing 10 % heat-inactivated fetal bovine serum, 0.25× brain heart infusion, 40 mM HEPES, 0.01 mg/ml hemin, 0.0002 % biotin, 100 IU/ml penicillin, 100 μg/ml streptomycin and 1× L-glutamine) at 26 °C and harvested in the mid-log phase. Electroporations were performed following the high-voltage protocol previously described [24]. Usually, 25–50 μg of test plasmid and cotransfection plasmid pLMRIB [25] were aliquoted into 4-mm gap cuvettes, and 500 μl of cells (2 × 10^8^ cells/ml) were added to the cuvette and mixed. The cells were electroporated twice at 1500 V and 25 μF (ECM 630 Electroporation System, BTX, Holliston, USA), pausing 10 s between pulses. Following electroporation, cells were transferred to 10 ml of BM medium and incubated at 26 °C, and total RNA was isolated 24 h post-transfection using TRI reagent (Sigma, St. Louis, USA).

### Bioinformatic analyses

Sequence information from different trypanosomatids was obtained from the NCBI (http://www.ncbi.nlm.nih.gov/) and TriTrypDB (Release 27) (http://tritrypdb.org/tritrypdb/) databases. BLAST searches were performed to obtain information for synteny maps and to search for U2-associated tRNA-like sequences. Alignments were generated using the MUSCLE (http://www.ebi.ac.uk/Tools/msa/muscle/) [26] and DNAMAN (version 6, Lynnon Corporation, Quebec, Canada) programs and corrected manually. Secondary structure analysis was performed using the RNAfold Web Server (http://rna.tbi.univie.ac.at/cgi-bin/RNAfold.cgi) [27] with default parameters. Graphical representations of patterns within multiple sequence alignments were generated with the WebLogo application (http://weblogo.threeplusone.com/).

### Primer extension analysis

Total RNA was analyzed by primer extension with 5' end-labelled synthetic DNA oligonucleotides as described previously [28]. The following oligonucleotides were used: 3'endLmjU2 (5'-GAA AAA AGG AGT TGC TCC CCT GGA AAC GTG-3'), complementary to U2 nt 122 to 151; LmjU2tag-Rev (5'-ATT AGA GTC GAG GTC AGA CC-3'), complementary to a 15-nt tag sequence inserted between U2 nt 83 and 84 (tag sequence is underlined); and 5'LmjU2DcajaB (5'-ATC CGG CCC TGG TCT CCA AA-3'), complementary to tRNA-like nt 58 to 77. In a typical experiment, 20 to 50 μg of total RNA were hybridized to 1 × 10^5^ cpm of γ-^32^P-labelled oligonucleotide at 55 °C for 90 min. The reaction volume was then increased to 50 μl by the addition of a solution containing 39.5 μl of RT buffer and 0.5 μl of SuperScript III Reverse Transcriptase (Invitrogen, Carlsbad, USA). The reaction was incubated at 55 °C for 60 min. Nucleic acids were precipitated and an aliquot of the products was separated on 8 % polyacrylamide-8 M urea gels and exposed to phosphor imaging plates. Autoradiograms were scanned on a Fujifilm FLA-5000 system (Fuji Photo Film Co., Ltd., Tokyo, Japan). When the U2 promoter region was analyzed, bands corresponding to a non-specific product were used to normalize samples of the U2 tagged-specific primer extension. Sequence ladders of U2, U2-tagged and tRNA-like sequences were obtained using a Sequenase 7-deaza-dGTP Sequencing Kit (USB, Cleveland, USA) as specified by the manufacturer. Constructs ptRNAU2 and pComp were used as templates in the sequencing reactions.

### 5'-RACE analysis

5' Rapid Amplification of cDNA Ends (5'-RACE) experiments were performed with 5 μg of total RNA from *L. major* with a kit from Thermo Scientific (Waltham, USA). First strand cDNA was synthesized with primer 3'U2GST1 (5'-AAC GTG GAA CTC CAA GGA AA-3'), and PCR amplifications were performed with nested primer 3'U2GST2 (5'-CCT TGA GTT GTA ATT TCT AT-3') and nested Abridged Anchor Primer, AAP (5'-GGC CAC GCG TCG ACT AGT ACG GGI IGG GII GGG IIG-3'). The nested PCR products were cloned into the pGEM-T Easy vector (Promega, Fitchburg, USA) and sequenced.

### RT-PCR analysis

U2 snRNA transcription termination sites were mapped by poly(A) tailing of total RNA, as previously reported [29]. The cDNA was prepared with oligonucleotide Nested(dT) (5'-CCT CTG AAG GTT CAC GGA TCC ACA TCT AGA TTT TTT TTT TTT TTT TTT VN-3'). The first PCR was performed with primers U2Sa-5' (5'-ATA TCT TCT CGG CTA TTT AGC-3') and B1 (5'-CCT CTG AAG GTT CAC GGA T-3'), and the second PCR was done with primers U2qFw2 (5'-CTA TTT AGC TAA GAT CAT GTT TAT A-3') and B2 (5'-CAC GGA TCC ACA TCT AGA T-3'). The final PCR products were cloned into the pGEM-T Easy vector (Promega) and sequenced.

### Mapping of pseudouridines

A primer extension-based pseudouridylation assay was carried out as previously described [30, 31]. Briefly, RNA from 5 × 10^8^ cells was treated with *N*-cyclohexyl-*N*'-β-(4-methylmorpholinium)ethyl-carbodiimide *p-*tosylate (CMCT) at 37 °C for 20 min in 50 μl of CMCT buffer (50 mM bicine, pH 8.0, 7 M urea and 4 mM EDTA) containing 170 mM CMCT reagent. To remove CMCT groups, the CMCT-treated RNA was subjected to alkali hydrolysis with 80 μl of Na_2_CO_3_ (50 mM, pH 10.4) at 37 °C for 2 h. One control experiment was done without CMCT treatment, and another one without alkali hydrolysis. The treated RNA (25 to 50 μg) was used as template in primer extension analysis with the 3'endLmjU2 oligonucleotide. In this method, the reverse transcriptase stops 1 nt before the modified base.

### Plasmid constructs

To obtain the plasmids used in this work, first a 429-bp fragment containing the U2 snRNA gene and 292 nt from its 5'-flanking sequence, was amplified by PCR with primers 5'LmjtRNAala (5'-TGA AAA AGT TGG AGA AGT TG-3') and 3'U2Lmjend (5'-TCC CCT GGA AAC GTG GAA CT-3'). The PCR product was cloned into the pGEM-T vector (Promega) to obtain plasmid ptRNAU2. This construct was used as template in a site-directed mutagenesis assay in which a 15-bp tag sequence was inserted between positions 83 and 84 of the U2 snRNA gene with oligonucleotides 5'U2oligoTag (5'-GCC TTC GGG CAA AGG TCT GAC CTC GAC TCT AAT AGA AAT TAC AAC-3') and 3'U2oligoTag (5'-GTT GTA ATT TCT ATT AGA GTC GAG GTC AGA CCT TTG CCC GAA GGC-3'). The resultant plasmid was called ptRNAU2M. This construct lacked the T tract located downstream of the U2 gene. Thus, to add the transcription termination signal, a 473-bp DNA fragment from ptRNAU2M was amplified with oligonucleotides LmjtRNAala-XmaI-F (5'-ACC CGG GTG AAA AAG TTG GAG AAG TTG-3') and endLmjU2-XbaI-R (5'-ATC TAG AGA AAA AAG GAG TTG CTC CCC TGG AAA CGT G-3') and cloned into the pGEM-T Easy vector (Promega), generating construct pComp. To obtain the vectors that contain sequential deletions of the U2 snRNA 5'-flanking sequence, several DNA fragments were amplified from pComp. The same reverse oligonucleotide (endLmjU2-XbaI-R) was used in all PCR reactions, together with the following forward oligonucleotides: U2tRNA-like-XmaI-F (5'-ACC CGG GAG GAA AAG ATG CTT TCG ACG AG-3') was used for pΔtRNA-Ala; LmjU2DcajaB-XmaI-F (5'-ACC CGG GAT CCC GCC CTG GTC TCC AAA-3') was used for pΔBox-B; LmjU2DcajaA-XmaI-F (5'-ACC CGG GGT AAG CGG CAC GGC AGT GAG-3') was employed for pΔBox-A; and primer U2Sa-XmaI-F (5'-ACC CGG GAT ATC TTC TCG GCT ATT TAG C-3') was used for pΔ-293/-1. All amplicons were cloned into the pGEM-T Easy vector (Promega). To obtain plasmids with base substitutions in the coding and 5'-flanking regions of the U2 snRNA, construct pComp was used as template in mutagenesis reactions performed with the following oligonucleotides: 5U2LmjmutFw (5'-TGG TAC TAA CAT ATC CAT GGA ACT ATT TAG CTA AGA T-3') and 5U2LmjmutRv (5'-ATC TTA GCT AAA TAG TTC CAT GGA TAT GTT AGT ACC A-3') for pBS + 6/+12; primers TLBTWU2LmjmutFw (5'-CAG GTA CCG TTT GGA TAG GAT CGC CCG ATT CCT TCG GGG TT-3') and TLBTWU2LmjmutRv (5'-AAC CCC GAA GGA ATC GGG CGA TCC TAT CCA AAC GGT ACC TG-3') for pBS-128/-138; oligonucleotides BalaU2LmjmutFw (5'-ATG CGG TAG GTA TTG TTG GAC GTA CCC AAC TTC TCC A-3') and BalaU2LmjmutRv (5'-TGG AGA AGT TGG GTA CGT CCA ACA ATA CCT ACC GCA T-3') for pBS-263/-269; primers AalaU2LmjmutFw (5'-ACC GCG TCG GGG ATG GAT ATC ATA GCC TAG AGC GAC CGC TTA-3') and AalaU2LmjmutRv (5'-TAA GCG GTC GCT CTA GGC TAT GAT ATC CAT CCC CGA CGC GGT-3') for pBS-219/-230; oligonucleotides BlikeU2LmjmutFw (5'-GCG GGA TTC CTT CGG TTG GAC GAC CTC GTC GAA AGC A-3') and BlikeU2LmjmutRv (5'-TGC TTT CGA CGA GGT CGT CCA ACC GAA GGA ATC CCG C-3') for pBS-150/-156; and primers AlikeU2LmjmutFw (5'-CGC GGC GTT TTT TTT GAT ACC ATC GCC TAC CGT TTG GAG ACC-3') and AlikeU2LmjmutRv (5'-GGT CTC CAA ACG GTA GGC GAT GGT ATC AAA AAA AAC GCC GCG-3') for pBS-105/-116. Finally, to generate pDBS that possesses a double mutation, plasmid pBS-105/-116 was used as template in a mutagenesis reaction with oligonucleotides BlikeU2LmjmutFw and BlikeU2LmjmutRv. All mutagenesis reactions were performed with a QuikChange Lightning Site-Directed Mutagenesis Kit (Agilent Technologies, Santa Clara, USA). In boxes A and B, the mutated nucleotides correspond to the most conserved positions, which have been shown to be important for Pol III transcription in other organisms [32, 33]. The identity of each insert was confirmed by sequencing using the T7 and SP6 oligonucleotides.

### Real-time quantitative PCR

Around 1 μg of DNase I-treated RNA from transiently transfected cells (from three independent transfections) was used as template for first strand cDNA synthesis using SuperScript™ III Reverse Transcriptase (Invitrogen) and 2 pmol of oligonucleotides 3'U2Lmjend or Alfa-tub-3' (5'-GTA GTT GAT GCC GCA CTT GAA G-3'). The cDNA was analyzed by real-time quantitative PCR (RT-qPCR) assays using the Platinum SYBR Green qPCR SuperMix-UDG kit (Invitrogen) in a Rotor-Gene 3000 cycler (Corbett Research, Mortlake, Australia) according to the manufacturer’s recommendations. All RT-qPCR reactions were performed at least in duplicate, using primers and conditions that were optimized to produce a single amplicon of the correct size. Each amplification product was analyzed for specificity by both agarose gel electrophoresis and melt curve analysis. Standard curves for primer pairs were derived from genomic and plasmid DNA dilution series and ranged in their r^2^ value from 0.98 to 1.0. PCR efficiencies were near to 100 % for the used genes, so the data were analyzed by the 2^-ΔΔCq^ method. For normalization of the data we used α-tubulin (*LmjF.13.0280*) as a reference gene, and all values were represented relative to data from cells transfected with plasmid pComp. The tagged-U2 snRNA was amplified with primers U2Sa-5' and LmjU2tag-Rev. The α-tubulin transcript was amplified with oligonucleotides Alfa-tub-5' (5'-AGA AGT CCA AGC TCG GCT ACA C-3') and a-tubLmjrvs (5'-GGT CGT AGA TGG CCT CAT TG-3'). To analyze RNA from cells transfected with pBS + 6/+12, the same conditions indicated above were performed, but using U2qFw2 as the sense primer in the PCR assays.

### Northern blot analysis

Total RNA (10–30 μg) was separated on 10 % polyacrylamide-8 M urea gels. After electrophoresis, RNA was transferred to Hybond N+ membranes (GE Healthcare, Little Chalfont, England) by electro-blotting using a Trans-Blot SD Semi-Dry Transfer Cell (Bio-Rad, Hercules, USA). The tRNA-Ala probe corresponds to the Ala-Rvs (5'-TCG ATC CCA ATA CCT ACC GC-3') oligonucleotide, the tRNA-like probe corresponds to the 5'LmjU2DcajaB oligonucleotide, the U2 snRNA probe to the 3'endLmjU2 oligonucleotide, and the tag probe to the LmjU2tag-Rev oligonucleotide. Primers were labelled with [γ-^32^P]ATP using T4 kinase. Hybridizations were performed in 6× SSPE (60 mM Na_2_HPO_4_, 0.9 M NaCl and 6 mM EDTA), 5× Denhardt’s reagent and 1 % SDS at 42 °C. Washing was carried out at 55 °C in 0.2× SSC and 0.1 % SDS.

## Results

### Genomic context of the U2 snRNA in *L. major* and other trypanosomatids

**Fig. 1:**
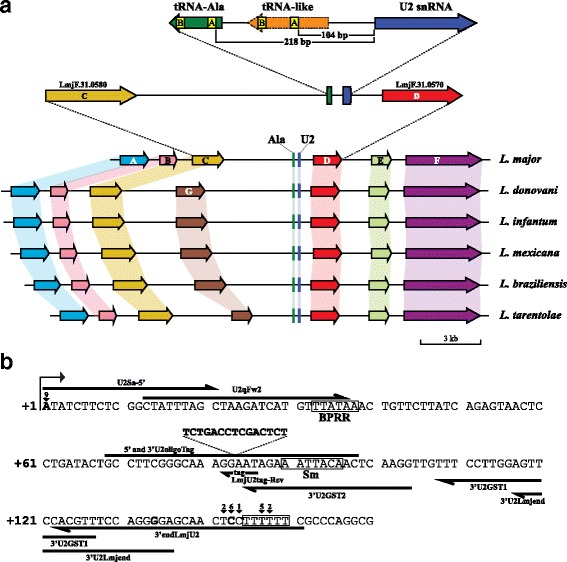
Genomic context and sequence of the U2 snRNA gene in *L. major.* **a** Synteny of U2 snRNA genes in different species of *Leishmania*. Orthologues genes are joined by colored lines. In *L. major*, gene A (*light blue*) corresponds to *LmjF.31.0600*, gene B (*pink*) to *LmjF.31.0590*, gene C (*mustard*) to *LmjF.31.0580*, gene D (*red*) to *LmjF.31.0570*, gene E (*light green*) to *LmjF.31.0560* and gene F (*purple*) to *LmjF.31.0550*. Gene G (*brown*) is absent in *L. major*, but corresponds to *LdBPK_310600.1* in *L. donovani*. Two enlargements of the *L. major* U2 snRNA gene are shown. On the top scheme are presented the U2 snRNA (*blue*), the tRNA-like (*orange*) and tRNA-Ala (*green*) genes. Boxes A and B within the tRNA-like and tRNA-Ala gene are shown in yellow. Distances between the U2 snRNA gene and boxes A from the tRNA-like (104 bp) and the tRNA-Ala gene (218 bp) are indicated. Figure is drawn to scale. **b** Sequence of the *L. major* U2 snRNA gene and 3'-flanking region. The A residue shown in bold type (position +1) corresponds to the TSS mapped by primer extension and 5'-RACE analyses (the arrowhead specify the number of clones found at that position). At the 3' end, the cluster of T nucleotides is shown in a box. The 3' termini mapped by RT-PCR are denoted with arrowheads, specifying the number of clones found at each position. The mature 3' terminus of the U2 snRNA (C at position +143) is shown in bold type. The missanotated 3' end of U2 at position 134 (G) is also shown in bold type. The locations of putative BPRR and Sm site are shown. The sequence of the tag, inserted between positions 83 (G) and 84 (A), is also indicated. The sequence and location of some of the oligonucleotides used in this study are shown. To facilitate the analysis of the results, the sequence is shown as the reverse-complement of the annotated sequence of chromosome 31 from the TriTrypDB database

The *L. major* genome contains a single copy of the U2 snRNA gene (*LmjF.31.snRNA.01*), which is embedded into an RNAP II polycistronic unit on chromosome 31 [34]. The U2 snRNA gene is flanked by two different amino acid transporter genes: *LmjF.31.0580* and *LmjF.31.0570* (Fig. 1a). A divergently-oriented tRNA-Ala gene (*LmjF.31.TRNAALA.01*) is located 211 bp upstream of the U2 snRNA gene (Fig. 1a). To determine the genomic context of U2 snRNA genes in other species of *Leishmania*, we examined the genome databases of *L. donovani* (strain BPK282A1) [35], *L. infantum* (JPCM5), *L. braziliensis* (M2904) [36, 37], *L. mexicana* (U1103) [37] and *L. tarentolae* (Parrot-TarII) [38]. Similarly to *L. major*, in all of the *Leishmania* species analyzed the U2 snRNA is encoded by a single-copy gene inserted into the same polycistronic unit (Fig. 1a), which is located on chromosome 31 in *L. donovani*, *L. infantum*, *L. braziliensis* and *L. tarentolae*, but on chromosome 30 in *L. mexicana* (as chromosomes 8 and 29 are fused in this species). Moreover, the divergently oriented tRNA-Ala gene is present in all of the species analyzed. Thus, synteny of the U2 snRNA locus is observed among *Leishmania* species. Interestingly, the genome of *L. major* lacks an extra amino acid transporter gene (Fig. 1a, gene G) that is present in all other species of *Leishmania*.

To extend the analysis of gene copy number of the U2 snRNA to other trypanosomatids, the genomic databases of members of the genera *Trypanosoma*, *Crithidia*, *Endotrypanum* and *Leptomonas* were examined. A single copy of the U2 snRNA gene is present in *T. brucei* (strains Lister 427, DAL972 and TREU927), *T. evansi* (STIB 805), *T. vivax* (Y486), *C. fasciculata* (Cf–Cl), *E. monterogeii* (LV88), *L. pyrrhocoris* (H10) and *L. seymouri* (ATCC 30220). Interestingly, two different strains of *T. cruzi* (CL Brener and Dm28_c) have three copies of the U2 snRNA gene. Thus, trypanosomatids possess only one copy of the U2 snRNA gene, with the exception of *T. cruzi* that contains three copies of this gene.

### Mapping of transcription initiation and termination sites in the U2 snRNA

**Fig. 2:**
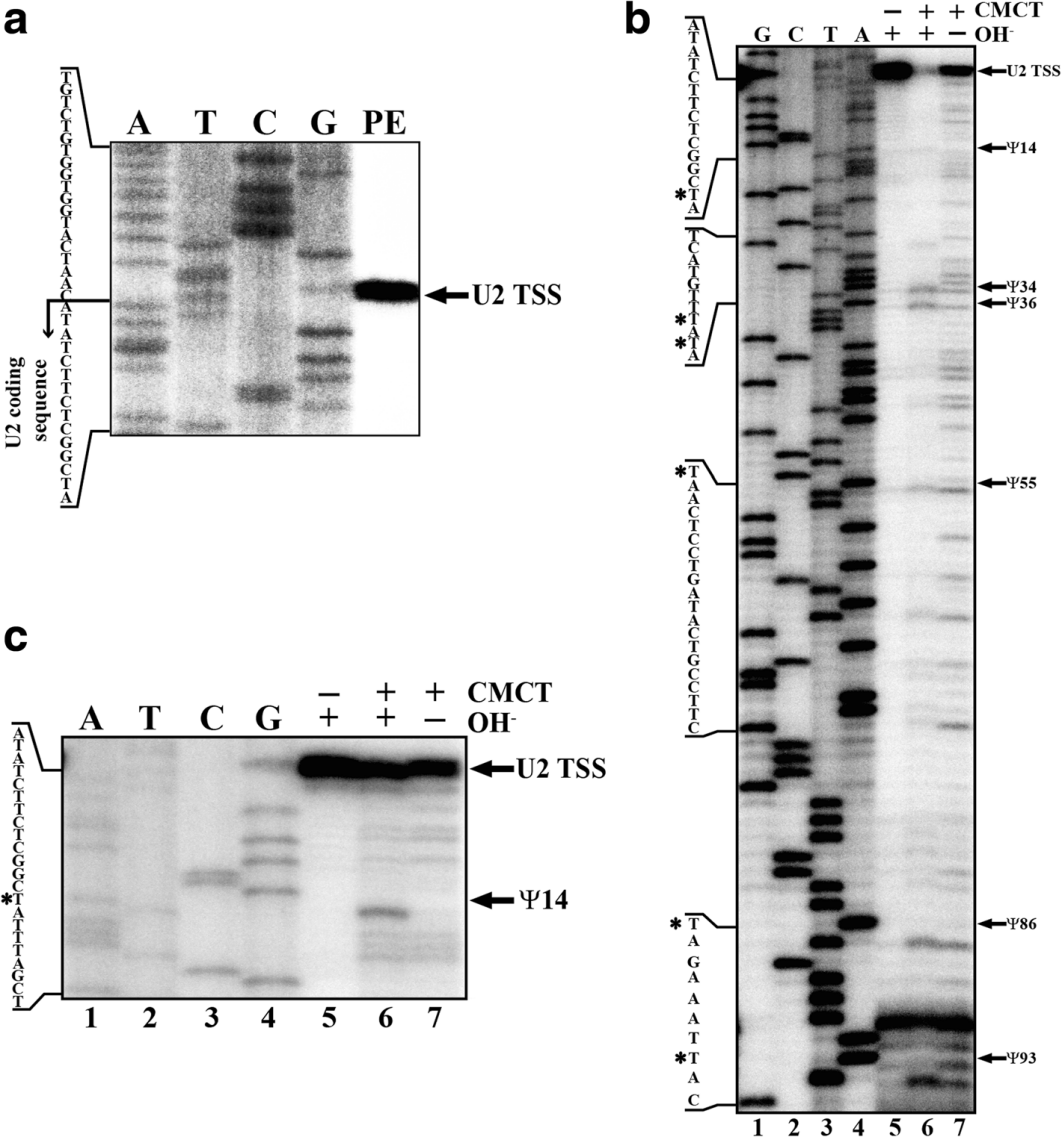
Primer extension analyses of the *L. major* U2 snRNA. **a** Mapping of the TSS of U2 snRNA in wild type promastigotes of *L. major*. Total RNA was subjected to primer extension with oligonucleotide 3'endLmjU2, and the products were separated on an 8 % polyacrylamide denaturing gel (Lane PE), along with a sequence ladder obtained with the same oligonucleotide (Lanes A, T, C and G). The arrow indicates the TSS, which corresponds to an A residue. Because the sequencing reactions were performed with 7-deaza dGTP (and the primer extension with dGTP), there is a size difference of about 1 bp between the sequence ladder and the primer extension product [48]. **b** Mapping of pseudouridines in U2 snRNA of *L. major*. Primer extension reactions were performed on total *L. major* RNA treated (+) or not (−) with CMCT for 20 min at 37 °C. CMCT-treated RNA was subjected to alkali hydrolysis (OH, +). One control experiment was done without alkali hydrolysis (OH, −). Primer extension and sequencing reactions were performed with oligonucleotide 3'endLmjU2. Arrows indicate the identified pseudouridines (Ψ) (reverse transcriptase stops 1 base before the pseudouridine). A partial sequence is shown on the left, indicating with asterisks the positions of the pseudouridylated nucleotides. **c** Primer extension-based pseudouridylation assay carried out as described in panel b. Only the upper part of the gel is shown

According to the TriTrypDB databases, the length of the *L. major* U2 snRNA gene is 134 bp (Fig. 1b). However, sequence comparisons (Additional file 1: Figure S1) suggest that the 3' end of the gene is missanotated. Thus, to determine the correct size of the U2 snRNA we mapped both the 5' and 3' termini of this snRNA. First, to locate the TSS, a primer extension assay was carried out. As shown in Fig. 2a, the TSS of U2 snRNA corresponds to an A residue (T in the complementary sequence), which is equivalent to the TSS previously identified in *T. cruzi*, *T. brucei* and *L. collosoma* [39, 40]. To corroborate the location of the TSS in the *L. major* U2 snRNA, a 5'-RACE assay was conducted. Analysis of nine cloned sequences confirmed that transcription of the U2 snRNA initiates at the A nucleotide (Fig. 1b). Thus, the TSS of the U2 snRNA is conserved in trypanosomatids.

To determine transcription termination sites of U2 snRNA in *L. major*, RT-PCR was performed with total RNA that was poly(A)-tailed *in vitro*. Transcription of U2 snRNA was found to end within a tract of six Ts located from 145 to 150 bp downstream of the TSS (Fig. 1b). In two of the clones analyzed, the poly(A) tail was added after the fourth T, and in five clones it was added after the third T residue. Trimming of these transcripts generate shorter RNA molecules, as one clone ended at nucleotide +144 (C), six clones ended at position +143 (C), and two clones at base +142 (U). Thus, it appears that during the processing of the 3' end of the U2 snRNA there are several populations of RNA with different 3' ends. Since most clones ended at the C residue located at position +143, we considered this nucleotide as the mature 3' terminus of the U2 snRNA in *L. major* (Fig. 1b). Consequently, by mapping both 5' and 3' ends of U2 snRNA in *L. major*, we have determined that the mature transcript is 143 nt long.

### Localization of pseudouridine residues in the U2 snRNA

Pseudouridylation of the U2 snRNA is essential for snRNP assembly and splicing in vertebrates and yeast [9]. To determine the presence of pseudouridine residues in the *L. major* U2 snRNA, total RNA was treated with CMCT followed by alkaline treatment and primer extension analysis. Considering that in this procedure the reverse transcriptase extension stops one base downstream of the CMCT-modified nucleotide, we found six pseudouridines in the U2 snRNA sequence at positions 14, 34, 36, 55, 86 and 93 (Fig. 2b, Lane 6). A control experiment was done without CMCT treatment to detect natural pauses of the reverse transcriptase, probably as a result of RNA degradation or the presence of stable RNA secondary structures (Fig. 2b, Lane 5). Another control without alkali hydrolysis was carried out to identify all the positions where the CMCT reagent was attached (Fig. 2b, Lane 7). In the experiment shown in Fig. 2b, the band that corresponds to pseudouridine at position 14 is very faint. However, in other experiments this band was clearly observed (Fig. 2c, Lane 6).

**Fig. 3:**
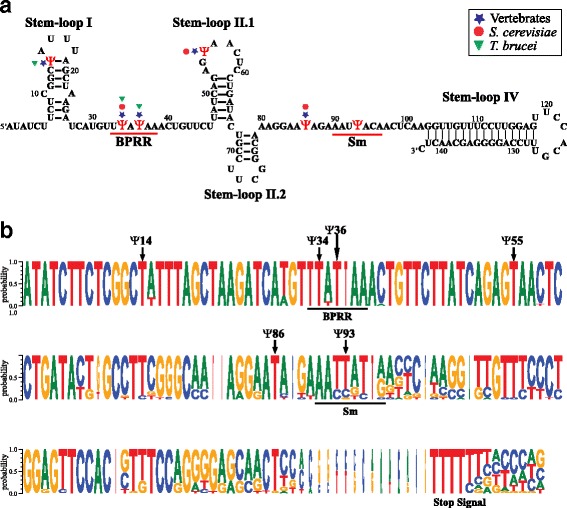
Sequence conservation and secondary structure of the U2 snRNA. **a** Predicted secondary structure of the *L. major* U2 snRNA. Stem-loops I, II.1, II.2 and IV are indicated. Also, BPRR and Sm site are shown. Pseudouridine residues (*red* Ψ) identified in this study are denoted. Pseudouridines conserved between *L. major* and vertebrates (*blue star*), *S. cerevisiae* (*red circle*) and *T. brucei* (*green triangle*) are also indicated. **b** Consensus sequence of the U2 snRNA gene in trypanosomatids. The sequences of the U2 snRNA genes and 3'-flaking regions from 21 species of trypanosomatids (see Additional file 1: Figure S1) were aligned with the DNAMAN software. Using the WebLogo application, the generated pattern was represented as a sequence logo by residue probabilities. BPRR, Sm site and stop signal are shown. The positions of the pseudouridine residues (Ψ) mapped in *L. major* are indicated with arrows

To determine if the pseudouridines are located in important functional domains of the U2 snRNA, the predicted secondary structure of the RNA molecule was generated and putative branch point recognition region (BPRR) and Sm binding sites were identified (Fig. 3a). Regarding the BPRR, the well-conserved metazoan sequence GUAGUA is replaced by UUAUAA in the *L. major* U2 snRNA (Fig. 3a). Also, the Sm site in the U2 snRNA, previously identified as AAUUACA (from nucleotides 90 to 96) (Fig. 3a), differs from the metazoan consensus sequence RAU_3-6_GR [41]. Interestingly, pseudouridines at positions 34 and 36 are located within the BPRR, and pseudouridine at base 93 is situated in the Sm site (Fig. 3a). It is worth noting that a pseudouridine in the Sm site has not been reported in any other organism.

As expected, the sequence of the U2 snRNA is conserved across trypanosomatids, as shown by a multiple sequence alignment (Additional file 1: Figure S1) and a sequence logo created with the WebLogo application [42] (Fig. 3b). Homology between the *L. major* U2 snRNA and its orthologues in other species of *Leishmania* is very high, ranging from 95.1 % identity with *L. braziliensis* to 98.6 % identity with *L. tarentolae*. Also, *L. major* U2 snRNA shows 69.4 % and 76.2 % identity to its orthologues in *T. cruzi* and *T. brucei*, respectively. Notably, we found that the U2 snRNA genes from all of the 21 species of trypanosomatids that we analyzed contain a T residue (U in the corresponding RNA sequence) in five of the six pseudouridylated positions identified in the *L. major* U2 snRNA (Fig. 3b and Additional file 1: Figure S1). The exception is position 93, which corresponds to a C or a G in five of the species analyzed. Thus, potentially pseudouridylated nucleotides are conserved in trypanosomatids.

### Characterization of the promoter elements in the *L. major* U2 snRNA

**Fig. 4:**
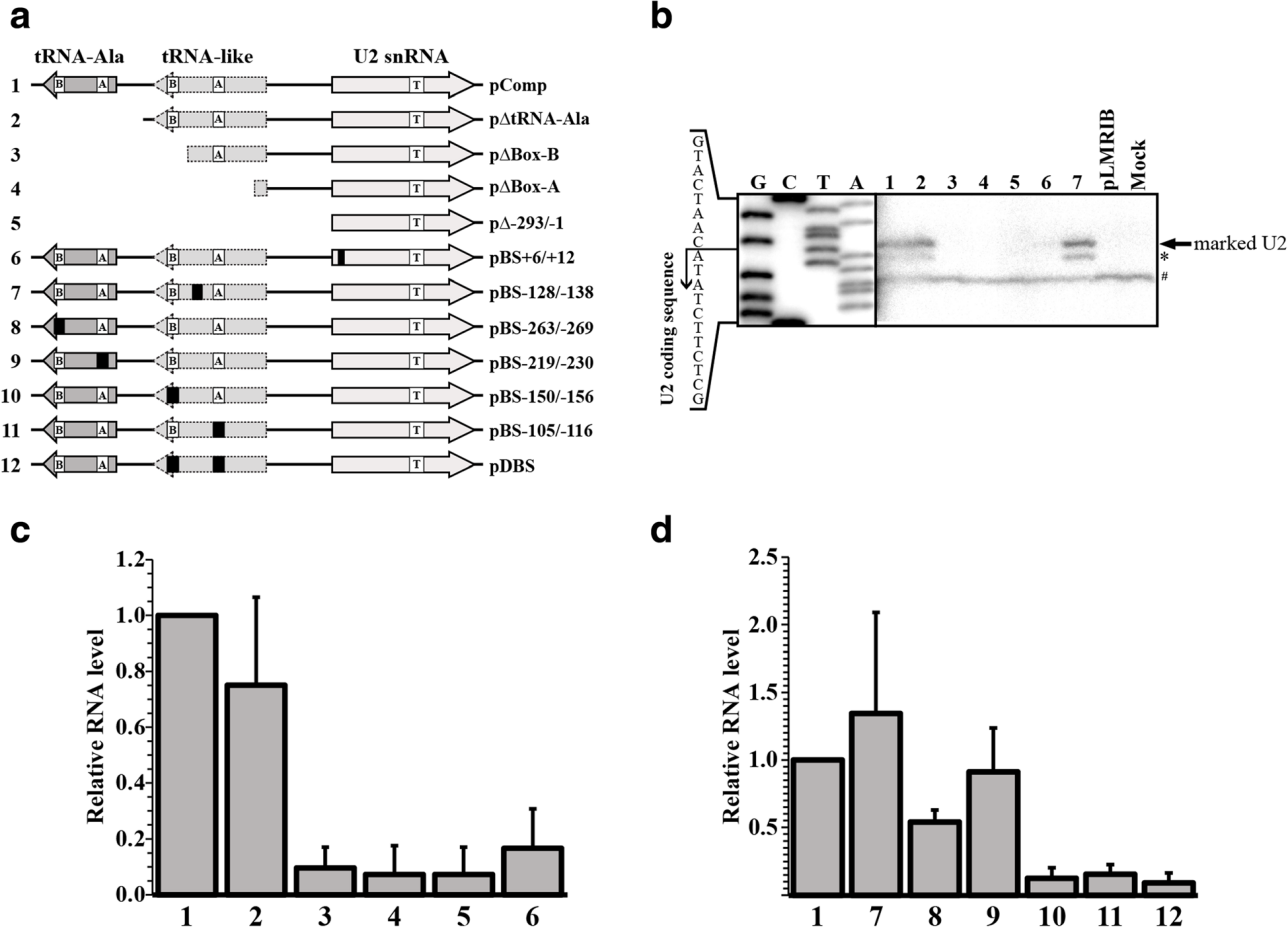
Identification of sequence elements required for the expression of the U2 snRNA. **a** Schematic representation of the constructs used for the transcription analysis. The location of the tag sequence (T in a box) is indicated in the U2 snRNA gene. Boxes A and B are shown in the tRNA-like and the tRNA-Ala gene. Black squares denote mutations by base substitutions. **b** Analysis of U2 snRNA promoter elements by primer extension assays. Constructs 1–7 were transfected into *L. major* cells and total RNA was obtained 24 h post-transfection. Primer extension analysis was performed with oligonucleotide LmjU2tag-Rev, which recognizes the tag sequence within the U2 exogenous transcripts. Products were separated on an 8 % polyacrylamide gel together with a DNA sequence ladder obtained with the same oligonucleotide. Lanes 1 to 7 correspond to RNA from cells transfected with vectors 1 to 7, respectively. Control experiments were carried out with RNA from mock-transfected cells (Mock), and from cells transfected with an unrelated vector (pLMRIB) [25]. **c** Transcriptional effect of progressive deletions of the 5'-flanking region of the U2 snRNA gene by RT-qPCR. The relative levels of the U2 snRNA were analyzed in cells transfected with constructs 1 to 6. **d** RT-qPCR assays to determine the effect of base substitutions in the expression of the U2 snRNA. The relative levels of U2 snRNA were analyzed in cells transfected with constructs 1 and 7–12. Error bars indicate standard deviations from three biological replicates

In *T. brucei* and *L. collosoma*, snRNA promoters consist of boxes A and B located inside tRNA or tRNA-like sequences found in the opposite direction of the snRNA. Interestingly, box A is consistently located 104 to 106 bp upstream of the snRNA. In *L. major*, box A of the tRNA-Ala gene is located 218 bp upstream of the U2 snRNA (Fig. 1a). However, we identified a tRNA-like sequence between the tRNA-Ala and the U2 snRNA genes (Fig. 1a). Boxes A and B from the tRNA-like sequence were located 105 and 150 bp upstream of the U2 snRNA, respectively. Thus, boxes A and B from the tRNA-like might control the transcription of the U2 snRNA. To test this hypothesis, we analyzed the transcription of several plasmid constructs after transient transfection of promastigotes. To identify transcripts produced by the transfected plasmids we marked the external U2 snRNA gene by insertion of a 15-nt tag sequence between positions +83 and +84 (Figs. 1b and 4a). Since no promoter sequences have been reported in this region, it is unlikely that the tag sequence could compromise the activity of a potential internal promoter element. Indeed, expression of tagged U2 snRNA was demonstrated by Northern blot (Additional file 2: Figure S2).

Thus, total RNA was extracted 24 h post-transfection and primer extension analyses were performed using an oligonucleotide complementary to the tag. As shown in Fig. 4b, with pComp (plasmid 1), which contains the complete 5' upstream region (tRNA-Ala and tRNA-like sequences) two primer extension products were detected (Fig. 4b, Lane 1). The longest product corresponds to the U2 snRNA TSS, while the other product was mapped to an A located at position +3 (marked with an asterisk in Fig. 4b) that might represent a reverse transcriptase stop. As expected, these bands were not present in the mock-transfected cells and cells transfected with a control vector (Fig. 4b, Mock and pLMRIB). Deletion of the tRNA-Ala gene (pΔtRNA-Ala) did not affect the abundance of the U2 snRNA bands (Fig. 4b, Lane 2). In contrast, elimination of box B from the tRNA-like (pΔBox-B, Lane 3), deletion of box A from the tRNA-like (pΔBox-A, Lane 4) and removal of the entire 5' flanking region (pΔ-293/-1, Lane 5) reduced the U2 snRNA transcripts to undetectable levels (Fig. 4b). Therefore, these results show that sequences within the tRNA-like are needed for transcription of the *L. major* U2 snRNA. To evaluate the presence of a possible internal promoter element in the snRNA gene, the sequence of the first nucleotides of the gene (from +6 to +12) were mutated (Additional file 3: Figure S3). In the primer extension analysis with this vector (pBS + 6/+12, Lane 6) the snRNA transcripts were strongly reduced, showing that the first bases of the U2 snRNA gene are required for expression of the gene. In contrast, mutation of the sequence located between boxes A and B in the tRNA-like did not affect the abundance of the U2 snRNA (pBS-128/-138, Lane 7) (Fig. 4b). A non-specific product that was observed in all the lanes was used as a control for RNA recovery (marked with a hash character in Fig. 4b).

To obtain a more precise determination of the abundance of the U2 snRNA transcripts, RNA from transiently-transfected cells was analyzed by RT-qPCR. As in the primer extension analysis, to discriminate between endogenous U2 and U2-tagged transcripts, an oligonucleotide complementary to the tag sequence was used. As shown in Fig. 4c, the U2 snRNA relative levels were severely reduced (by 90–93 %) when box B of the tRNA-like and sequences downstream were deleted (compare bar 1 to 3–5). Thus, these results are in agreement with the primer extension results (Fig. 4b, Lanes 3–5), and indicate that the tRNA-like is needed for the *in vivo* expression of the U2 snRNA. To analyze independently the transcriptional roles of boxes A and B from the tRNA-like, base substitutions were produced in these two elements (Additional file 3: Figure S3). We observed that mutations in box B (Fig. 4d, bar 10), box A (Fig. 4d, bar 11) and both boxes (Fig. 4d, bar 12) drastically diminished the U2 snRNA levels to 13, 16 and 9 %, respectively. By contrast, a slight increase in the levels of U2 snRNA was found with the control vector that bears a mutation in the region located between boxes A and B in the tRNA-like, where no promoter element is found (Fig. 4d, bar 7). In agreement with the primer extension analysis, mutation of the first nucleotides of the U2 snRNA gene diminished the RNA levels to 17 % of the value of the control (Fig. 4c, bar 6).

An unexpected result was that deletion of the tRNA-Ala gene, located upstream of the tRNA-like, produced a 25 % reduction in the levels of the U2 snRNA (Fig. 4c, bar 2). To further explore this effect, base substitutions in boxes A and B of the tRNA-Ala gene were generated and analyzed. Interestingly, when box B was mutated, the expression of the U2 snRNA was reduced to 54 % of the value for the control (Fig. 4d, bar 8). In contrast, mutations in box A from the tRNA-Ala gene have a slight impact on the abundance of the U2 snRNA (Fig. 4d, bar 9). Therefore, our results show that box B from the tRNA-Ala gene located upstream of the tRNA-like is important for transcription of the U2 snRNA in *L. major*.

### The tRNA-like sequence associated to the U2 snRNA is transcribed

**Fig. 5:**
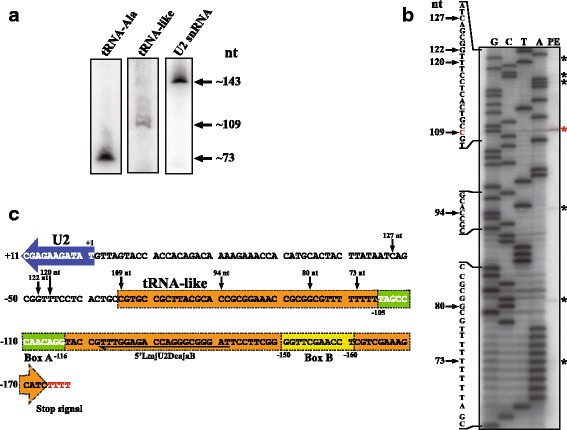
Transcription of the tRNA-like sequence associated to the U2 snRNA gene. **a** Northern blot analyses with specific probes to detect the tRNA-Ala (left image), tRNA-like (middle image) and U2 snRNA (right image). **b** Mapping of the 5' ends of tRNA-like molecules in wild type promastigotes of *L. major*. Total RNA was subjected to primer extension analysis with oligonucleotide 5'LmjU2DcajaB, and the products were separated on an 8 % polyacrylamide denaturing gel (Lane PE) together with a DNA sequencing ladder obtained with the same oligonucleotide (Lanes G, C, T and A). The primer extension products are marked with asterisks; the red asterisk denotes the most abundant product. Partial sequences of the tRNA-like are shown on the left, indicating with arrows the positions of the 5' termini and the expected size of tRNA-like molecules. **c** Sequence of the tRNA-like, which is boxed with dotted lines (*orange box* with an arrow indicating the direction of transcription). Primer extension products are marked with arrows, indicating the length of the transcript. The most abundant primer extension product was considered as the TSS (C, 109 nt). Boxes A and B are colored in green and yellow, respectively. The tRNA-like transcription stop signal is shown in red. The location of the 5'LmjU2DcajaB oligonucleotide is indicated. The first eleven nucleotides of the U2 snRNA gene are also shown. Sequence numbers are relative to the TSS (+1) from the U2 snRNA. To facilitate the analysis of the results, the sequence is shown as presented in the TriTrypDB database (which is the reverse-complement of the sequence shown in Fig. 1)

To determine whether the tRNA-like sequence linked to the *L. major* U2 snRNA gene is transcribed, a Northern blot experiment was performed with total RNA isolated from wild type *L. major* promastigotes. A band of around 109 nt was detected, indicating that the tRNA-like is transcribed in *L. major* (Fig. 5a, middle panel). Several larger and weaker bands were also observed, which may represent longer tRNA-like transcripts or unspecific bands. Control experiments to detect the U2 snRNA and the tRNA-Ala showed the expected bands of around 143 and 73 nt, respectively (Fig. 5a). Densitometry analysis of the observed bands showed that the tRNA-like is at least 10-fold less abundant than the U2 snRNA.

The TSS of the tRNA-like was mapped by primer extension (Fig. 5b). The most abundant band corresponded to a C residue (G in the complementary sequence) (labelled with a red asterisk in Fig. 5b) located 65 bases upstream of the U2 snRNA (Fig. 5c). If we assume that transcription of the tRNA-like ends in the T-tract located downstream of the gene (Fig. 5c), the tRNA-like transcript would be 109 nt long, which is in agreement with the size observed in the Northern blot (Fig. 5a). The smaller bands detected in the primer extension analysis (73, 80 and 94 nt) might represent reverse transcriptase stops, while the larger bands (120, 122 and 127 nt) could denote alternative TSS (black asterisks in Fig. 5b, and black arrows in Fig. 5c).

### Sequence analysis of boxes A and B from tRNA-like sequences associated to U2 snRNA genes

**Fig. 6:**
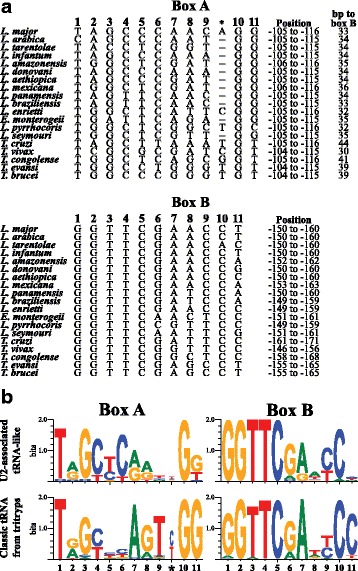
Analysis of boxes A and B from tRNA-like sequences associated to U2 snRNA genes. **a** Alignment of boxes A and B from the tRNA-like sequences of the following species of trypanosomatids: *L. major* (Friedlin), *L. arabica* (LEM1108), *L. tarentolae* (Parrot-TarII), *L. infantum* (JPCM5), *L. amazonensis* (MHOMBR/71973/M2269), *L. donovani* (BPK282A1), *L. aethiopica* (L147), *L. mexicana* (U1103), *L. panamensis* (MHOM/COL/81/L13), *L. braziliensis* (M2904), *L. enriettii* (LEM3045), *E. monterogeii* (LV88), *L. pyrrhocoris* (H10), *L. seymouri* (ATCC 30220), *T. cruzi* (CL Brener Non-Esmeraldo-like, copy of the chromosome 23), *T. vivax* (Y486), *T. congolense* (IL3000), *T. evansi* (STIB 805) and *T. brucei* (DAL972). All sequences were obtained from the TriTrypDB databases. In box A, some tRNA-like sequences contain an extra nucleotide between positions 9 and 10 (marked with an asterisk). For each box, its position in relation to the TSS of the associated U2 snRNA is indicated. The distance between boxes A and B is also shown. **b** Consensus sequences of boxes A and B from tRNA-like regions associated to U2 snRNA genes in trypanosomatids. The alignments shown in panel A were used to generate sequence logos with the WebLogo application (top logos). Additional sequence logos were obtained for boxes A and B from all of the 258 classic tRNAs found in TriTryps [43] (lower logos). All four sequence logos are presented by their information content

Sequence analysis of boxes A (TAG CCC AAC AGG) and B (GGT TCG AAC CT) from the tRNA-like associated to the *L. major* U2 snRNA revealed that they are similar to boxes A (TAG CTC AGA TGG) and B (GGA TCG ATA CC) from the tRNA-Ala gene. To compare these sequence elements across trypanosomatids, we first searched for boxes A and B in tRNA-like sequences associated to U2 snRNA genes in the genome databases of 18 additional species of trypanosomatids (Fig. 6a). A tRNA-like sequence with boxes A and B was found in all the species analyzed (Fig. 6a); and sequence logos were obtained for both sequence elements (Fig. 6b, upper logos). For comparison, sequences of boxes A and B from all of the 258 classic tRNAs found in TriTryps [43] were aligned and logo sequences were generated (Fig. 6b, lower logos). Regarding tRNA-like sequences, analysis of the sequence logo from box A showed a high conservation of most bases, with the exception of the position labelled with an asterisk (which is absent in most species analyzed) and base 9 (Fig. 6b, upper logo). Box B is even more conserved, as positions 1 to 5 (GGTTC) are present in all the sequences, and the rest of the bases are conserved, with the exception of positions 8 and 11 (Fig. 6b, upper logo). In general, high sequence conservation was observed when comparing boxes B from tRNA-like sequences and classic tRNA genes, since position 11 is the only one that is not conserved (Fig. 6b, right logos). On the other hand, boxes A are not as highly conserved as boxes B, since positions 5, 6, 7, 9, 11 and (*) differ between tRNA-like and classic tRNA genes (Fig. 6b, left logos).

It is interesting to note that a distance of 104 bp separates the U2 snRNA gene and box A from the tRNA-like sequence in most trypanosomatids (Fig. 6a). Moreover, the distance that separates box A from box B in the tRNA-like sequences is conserved in *Leishmania*, fluctuating between 33 bases (in *L. major* and *L. braziliensis*) and 36 bases (in *L. mexicana*). In contrast, this distance is not conserved in tRNA-like sequences from trypanosomes, as it varies from 30 bases (*T. vivax*) to 44 bases (*T. cruzi*) (Fig. 6a). Thus, our analysis shows that, while boxes A are more divergent in sequence, their position in relation to the U2 snRNA gene is almost invariable. On the other hand, the sequences of boxes B are highly conserved but their distance to box A (or to the snRNA gene) is variable across trypanosomatids.

## Discussion

In this work we have studied the sequence, structure and genomic context of the U2 snRNA gene in *Leishmania* and other trypanosomatids, and have identified the sequences that drive transcription of the U2 snRNA in *L. major*. Although in general the sequence of the U2 snRNA is highly conserved in trypanosomatids, the sequence and length of its 3' end are variable (Fig. 3b and Additional file 1: Figure S1). Thus, we mapped both 5' and 3' ends of U2 snRNA in *L. major* and determined that the mature transcript is 143 nt long (Fig. 1b), which is similar to the 143 and 145 nt reported for the U2 snRNA in *T. cruzi* and *T. brucei*, respectively [44, 45].

The predicted secondary structure of the U2 snRNA in *L. major* (Fig. 3a) resembles the structure reported for *T. brucei*, as they both lack stem-loop III (Fig. 3a). Out of the six pseudouridine residues that we found in the *L. major* U2 snRNA (Fig. 2b), five are also present in vertebrates (positions 14, 34, 36, 55 and 86 in *L. major*), three have been found in *S. cerevisiae* (positions 34, 55 and 86) [8, 9], and three are present in *T. brucei* (positions 14, 34 and 36) [31] (Fig. 3a). It is worth noting that the only pseudouridine that is conserved in all of the organisms analyzed is the one located at position 34, which is part of the motif involved in the branch site recognition (Fig. 3a). Pseudouridine at position 36 is also located in the BPRR. Interestingly, the *L. major* U2 snRNA contains one pseudouridine that has not been reported in other organisms, which is located within the Sm site at position 93 (Fig. 3a). Thus, it is likely that the pseudouridines we mapped in the *L. major* U2 snRNA are important for snRNP assembly and trans-splicing.

A tRNA-like was found between the U2 snRNA and the tRNA-Ala genes in *L. major* (Fig. 1a). Primer extension and RT-qPCR analyses showed that transcription of the U2 snRNA gene is dependent on boxes A and B located inside the tRNA-like sequence, since deletion or mutation of these elements strongly reduced the level of the snRNA. Also, an intragenic promoter element was found close to the 5' end of the U2 snRNA gene (Fig. 4). Similar promoter elements drive the expression of the U2 snRNA gene in *T. brucei* [17]. Moreover, boxes A and/or B located in tRNA-like or tRNA genes have been shown to control the expression of the U1, U3 and U6 snRNA genes in *T. brucei*, the U4 snRNA gene in *L. collosoma* and 7SL RNA genes in *T. brucei* and *L. collosoma* [18, 19, 22, 23, 46]. However, unlike any other snRNA or 7SL RNA genes in trypanosomatids, our data show that box B from the upstream tRNA-Ala also participates in the regulation of transcription of the U2 snRNA gene in *L. major*. While mutation of box B from the tRNA-like reduced the U2 snRNA expression by 87 %, base substitutions in box B from the tRNA-Ala decreased the levels of the U2 snRNA by 46 % (Fig. 4d, compare bars 8 and 10). Thus, it is possible that box B from the tRNA-Ala participates in the fine-tuning of transcription of the *L. major* U2 snRNA.

At present it is not known how boxes A and B from the associated tRNA or tRNA-like control transcription of the snRNA. It has been proposed that transcription factors binding to boxes A and B would be shared by the snRNA and tRNA (or tRNA-like) genes. Alternatively, boxes A and B could regulate snRNA expression by an indirect mechanism involving the binding of transcription factors to the tRNA gene, which in turn could generate changes in chromatin structure that would allow transcription of the snRNA [21]. In this regard, very little is known about RNAP III transcription factors in trypanosomatids. From the two transcription factors required for the expression of tRNA genes, TFIIIC and TFIIIB, only the latter has been identified in trypanosomatids [47]. Thus, it is possible that the highly-transcribed tRNA-Ala gene, and the tRNA-like sequence, might bind abundant transcription factors and produce an open chromatin conformation that could facilitate transcription of the U2 snRNA in *L. major*.

Our results showed that the tRNA-like associated to the U2 snRNA is transcribed, generating a major transcript of around 109 nt (Fig. 5a, middle panel). Transcription of tRNA-like sequences associated with the *T. brucei* U2 snRNA and the *L. collosoma* U4 snRNA have also been demonstrated, producing transcripts of 89 and 90 bases, respectively [20, 23]. Densitometric analysis indicated that the tRNA-like transcript is at least 10-fold less abundant than the U2 snRNA. Thus, if the U2 snRNA and the tRNA-like are transcribed together and at the same rate, the tRNA-like should be less stable than the U2 snRNA. The predicted secondary structure of the tRNA-like was very different from the expected for a tRNA, as it does not fold into the classical cloverleaf structure (Additional file 4: Figure S4). Consequently, it is possible that this atypical secondary structure might contribute to the apparent instability of the tRNA-like transcript. Other possibility is that the transcription rate in the two directions is different and, accordingly, lower amounts of tRNA-like are produced.

Our data show that all of the *Leishmania* species analyzed contain a single U2 snRNA gene, which is highly syntenic (Fig. 1a). Similarly, in other trypanosomatids the U2 snRNA is encoded by a single-copy gene, with the exception of *T. cruzi* and *L. collosoma* that contain three genes [39]. In *T. cruzi*, only one of the U2 snRNA genes is transcribed [39]. Interestingly, sequence analysis allowed us to determine that the two *T. cruzi* U2 snRNA genes that are not expressed possess a box A in the associated tRNA-like sequence, but do not contain a box B (Additional file 5: Figure S5). Thus, the absence of box B in the neighbor tRNA-like sequence might contribute to the silencing of two U2 snRNA genes in *T. cruzi*. Remarkably, from all the trypanosomatids with genome sequences available, *C. fasciculata* is the only organism that does not have a tRNA-like associated with the U2 snRNA, as it contains a tRNA-Ala gene whose box A is located 105 bp upstream of the U2 snRNA gene (Additional file 6: Figure S6).

## Conclusions

In the present study we have demonstrated that transcription of the *L. major* U2 snRNA gene is dependent on boxes A and B from the associated tRNA-like and a sequence located at the 5' end of the U2 snRNA. Notably, unlike any other snRNA or 7SL RNA genes in trypanosomatids, box B from the upstream tRNA-Ala is also important for the expression of the *L. major* U2 snRNA gene. Thus, the U2 snRNA gene promoter shows unique features in *L. major*. Also, we have mapped several pseudouridines in the *L. major* U2 snRNA gene, including one located in the Sm binding site that has not been reported in any other organism. Future studies will explore the role that pseudouridines might play in splicing and snRNP assembly in *Leishmania*.

## Abbreviations

5'-RACE, 5' Rapid Amplification of cDNA Ends; BPRR, branch point recognition region; CMCT, N-cyclohexyl-N'-β-(4-methylmorpholinium)ethyl-carbodiimide p-tosylate; mRNA, messenger RNA; RNAP, RNA polymerase; RT-qPCR, real-time quantitative polymerase chain reaction; snRNA, small nuclear RNA; tRNA, transfer RNA; TSS, transcription start site

## Additional files

Additional file 1: Figure S1. Sequence alignment of U2 snRNA genes from trypanosomatids. Sequences were obtained from the TriTrypDB databases of the following species of trypanosomatids: *L. major* Friedlin (Lmj), *L. arabica* LEM1108 (Lar), *L. tarentolae* Parrot-TarII (Ltr), *L. infantum* JPCM5 (Lin), *L. amazonensis* MHOMBR/71973/M2269 (Laz), *L. donovani* BPK282A1 (Ldn), *L. aethiopica* L147 (Lae), *L. mexicana* U1103 (Lmx), *L. panamensis* MHOM/COL/81/L13 (Lpn), *L. braziliensis* M2904 (Lbr), *L. enriettii* LEM3045 (Len), *E. monterogeii* LV88 (Emg), *C. fasciculata* Cf-Cl (Cfs), *L. pyrrhocoris* H10 (Lpy), *L. seymouri* ATCC 30220 (Lsy), *L. collosoma* (unspecified strain) [39] (Lcl), *T. cruzi* CL Brener Non-Esmeraldo-like, copy of the chromosome 23 (Tcr), *T. vivax* Y486 (Tvx), *T. congolense* IL3000 (Tcn), *T. evansi* STIB 805 (Tev) and *T. brucei* DAL972 (Tbr). The alignment was obtained with the DNAMAN software, and manually corrected. Arrows indicate the identified pseudouridines (Ψ) in the *L. major* U2 snRNA (this work). BPRR and Sm sites are also indicated. The 3' ends (C residues) that have been experimentally mapped are shown in red font for *L. major* (this work), *T. cruzi* [45] and *T. brucei* [44]. The T-tract length is indicated for each species. Sequence numbers are relative to the TSS (+1) from the U2 snRNA. This alignment was used to generate the sequence logo depicted in Fig. 3b. (PDF 2137 kb) Additional file 2: Figure S2. Northern blot analysis of the tagged U2 snRNA. The experiment was performed with total RNA from cells transfected with construct 1 (pComp), construct 6 (pBS + 6/+12), the unrelated vector pLMRIB or mock-transfected cells. The probe was the oligonucleotide LmjU2tag-Rev, which specifically recognizes the tag sequence. (TIF 807 kb) Additional file 3: Figure S3. Mutations introduced in the tRNA-Ala/U2 snRNA locus. For each vector, sequence elements are indicated inside colored boxes: tRNA-Ala in green, tRNA-like in orange and U2 snRNA in blue. Boxes A and B are also indicated. The tag sequence, inserted within the U2 snRNA gene, is also shown. Base substitutions are indicated in red font. (PDF 3122 kb) Additional file 4: Figure S4. Predicted secondary structure of the tRNA-Ala (panel a) and the tRNA-like associated to the *L. major* U2 snRNA gene (panel b). The minimum free energy structures are shown. The color scale indicates low (*blue*) to high (*red*) probabilities of base pairing. (PDF 355 kb) Additional file 5: Figure S5. Sequence comparisons of U2 snRNA genes and flanking regions from *T. cruzi* (CL Brener Non-Esmeraldo-like). Sequences from the genes located on chromosomes 23, 37 and 6 are shown. The U2 snRNA gene from chromosome 23 is presented in blue font. The position of boxes A and B is indicated. Sequence numbers are relative to the TSS (+1) from the U2 snRNA. (PDF 1404 kb) Additional file 6: Figure S6. Genomic context of U2 snRNA genes in trypanosomatids. Schematic representations of the U2 snRNA loci from *Leishmania* spp., *E. monterogeii* (LV88), *L. seymouri* (ATCC 30220), *C. fasciculata* (Cf-Cl), *T. cruzi* (CL Brener Non-Esmeraldo-like, copy of the chromosome 23) and *T. brucei* (DAL972). All of the U2 snRNA genes contain divergently-oriented boxes A and B at conserved distances (around 104 bases), which are contained in tRNA-like sequences. The exception is *C. fasciculata*, where boxes A and B are located inside a tRNA-Ala gene. In *T. cruzi*, a tRNA-Arg gene is located downstream of the U2 snRNA gene. Figure is drawn to scale. (TIF 268 kb)
