# Supplementary figures and images for: Transcription of Leishmania major U2 small nuclear RNA gene is directed by extragenic sequences located within a tRNA-like and a tRNA-Ala gene
Source: Parasit Vectors. 2016 Jul 19;9:401. doi: 10.1186/s13071-016-1682-3 (PMC4950102; doi:10.1186/s13071-016-1682-3)

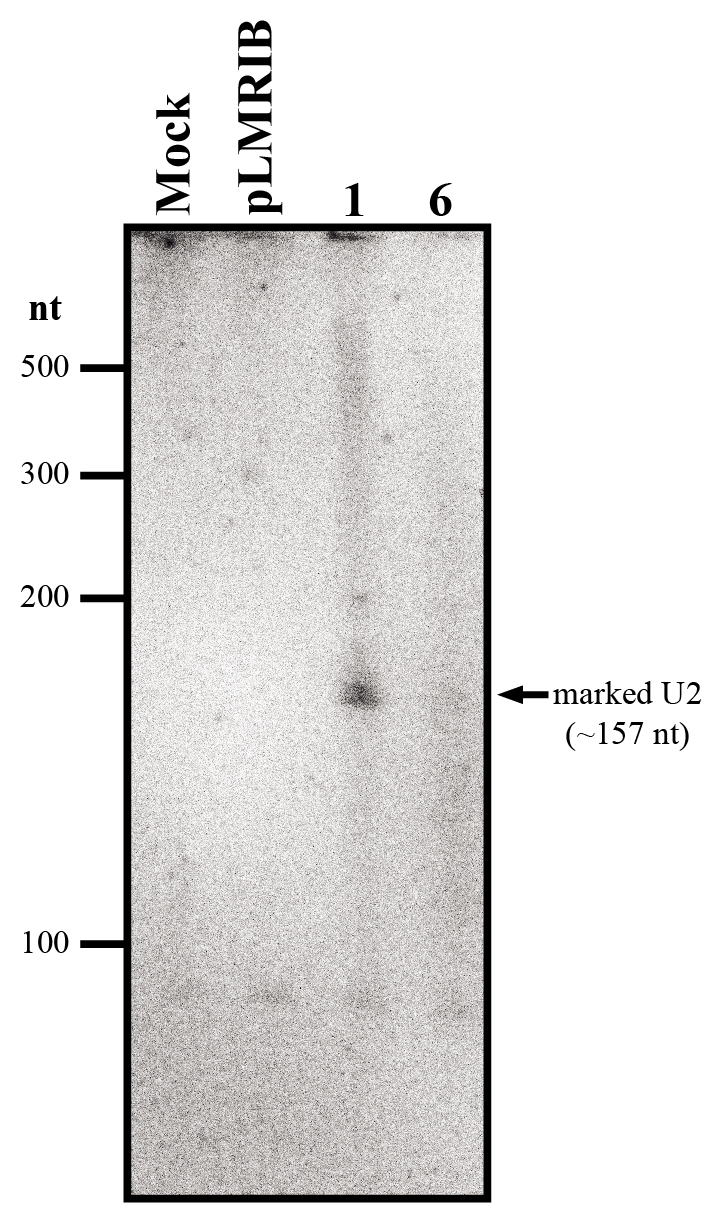

Supplement: Additional file 2: Figure S2. — Northern blot analysis of the tagged U2 snRNA. The experiment was performed with total RNA from cells transfected with construct 1 (pComp), construct 6 (pBS + 6/+12), the unrelated vector pLMRIB or mock-transfected cells. The probe was the oligonucleotide LmjU2tag-Rev, which specifically recognizes the tag sequence. (TIF 807 kb) [file 13071_2016_1682_MOESM2_ESM.tif]

**a****tRNA-Ala (73 nt)**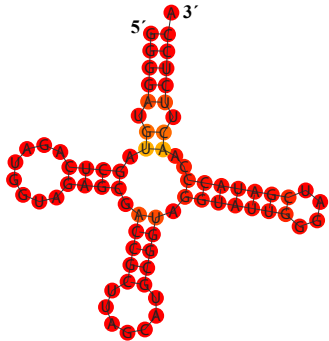**b****tRNA-like (109 nt)**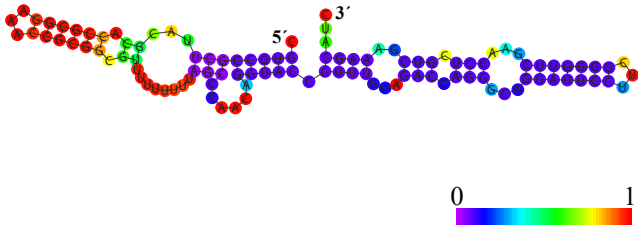

Supplement: Additional file 4: Figure S4. — Predicted secondary structure of the tRNA-Ala (panel a) and the tRNA-like associated to the L. major U2 snRNA gene (panel b). The minimum free energy structures are shown. The color scale indicates low (blue) to high (red) probabilities of base pairing. (PDF 355 kb) [file 13071_2016_1682_MOESM4_ESM.pdf]

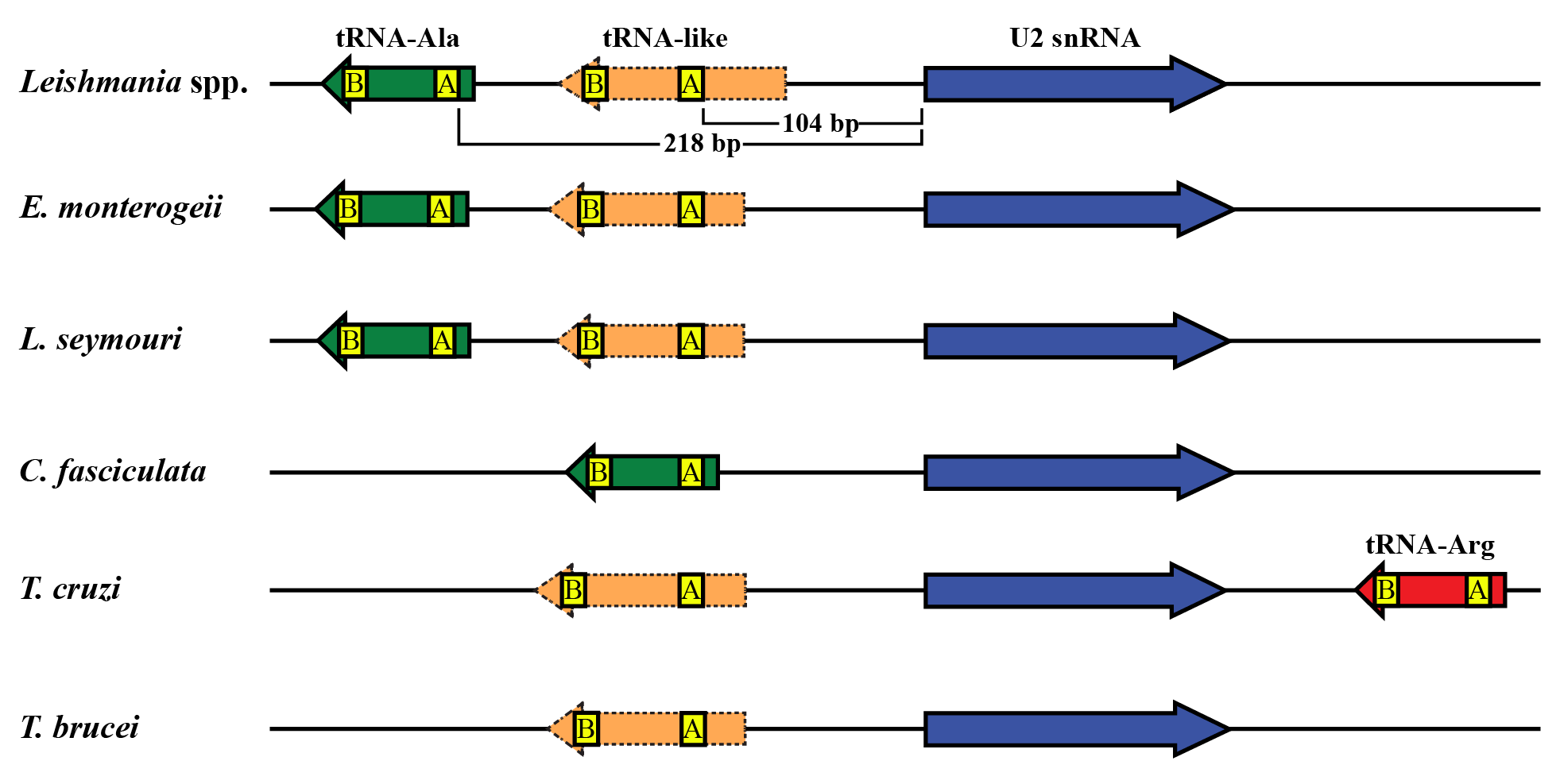

Supplement: Additional file 6: Figure S6. — Genomic context of U2 snRNA genes in trypanosomatids. Schematic representations of the U2 snRNA loci from Leishmania spp., E. monterogeii (LV88), L. seymouri (ATCC 30220), C. fasciculata (Cf-Cl), T. cruzi (CL Brener Non-Esmeraldo-like, copy of the chromosome 23) and T. brucei (DAL972). All of the U2 snRNA genes contain divergently-oriented boxes A and B at conserved distances (around 104 bases), which are contained in tRNA-like sequences. The exception is C. fasciculata, where boxes A and B are located inside a tRNA-Ala gene. In T. cruzi, a tRNA-Arg gene is located downstream of the U2 snRNA gene. Figure is drawn to scale. (TIF 268 kb) [file 13071_2016_1682_MOESM6_ESM.tif]
