# Supplementary material for: Transcription of Leishmania major U2 small nuclear RNA gene is directed by extragenic sequences located within a tRNA-like and a tRNA-Ala gene
Source: Parasit Vectors. 2016 Jul 19;9:401. doi: 10.1186/s13071-016-1682-3 (PMC4950102; doi:10.1186/s13071-016-1682-3)
Supplement: Additional file 3: Figure S3. — Mutations introduced in the tRNA-Ala/U2 snRNA locus. For each vector, sequence elements are indicated inside colored boxes: tRNA-Ala in green, tRNA-like in orange and U2 snRNA in blue. Boxes A and B are also indicated. The tag sequence, inserted within the U2 snRNA gene, is also shown. Base substitutions are indicated in red font. (PDF 3122 kb) [file 13071_2016_1682_MOESM3_ESM.pdf]

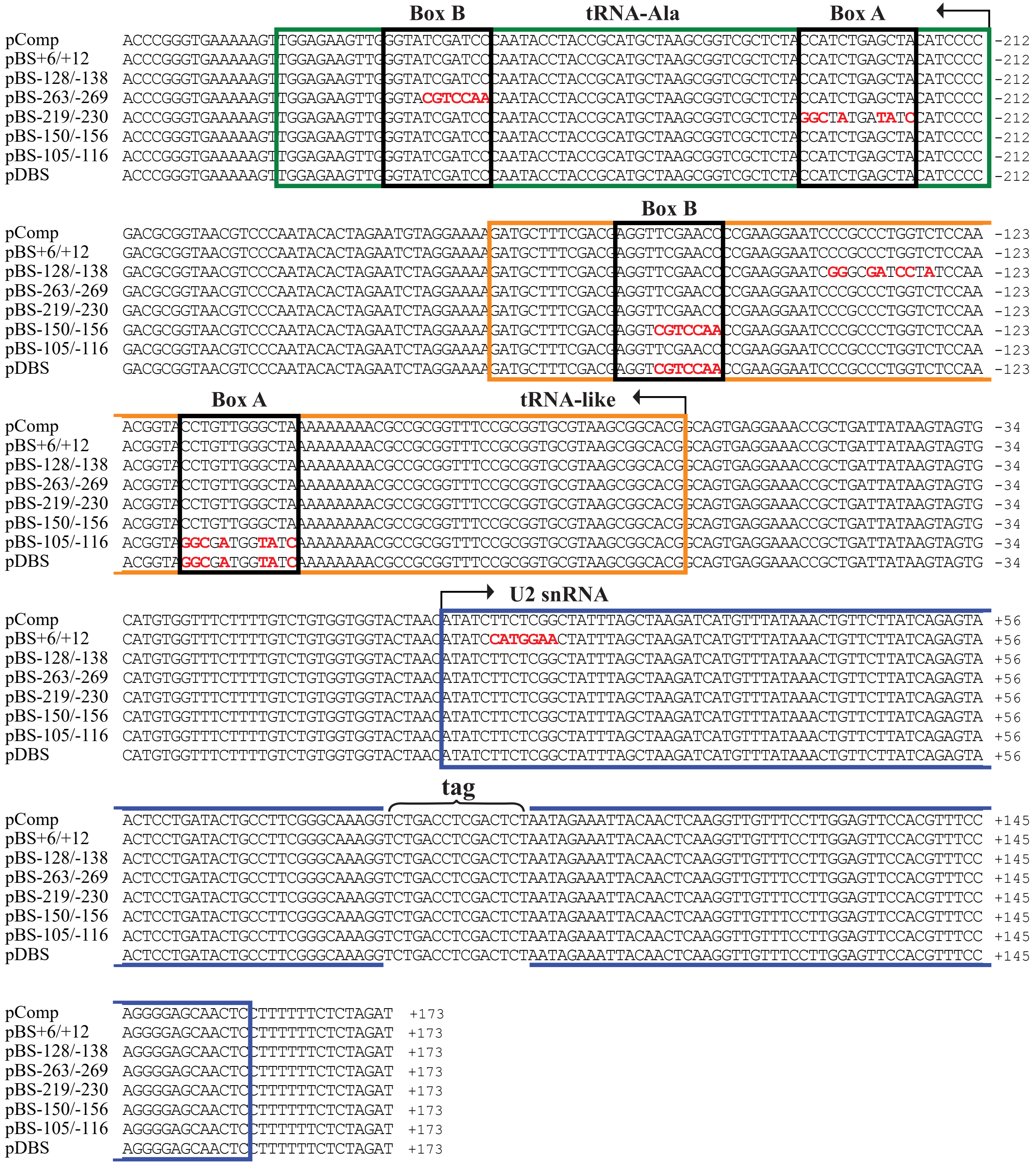

Box B
tRNA-Ala
Box A
pComp ACCCGGGTGAAAAAGTTGGAGAAGTTGGGTATCGATCCCAATACCTACCGCATGCTAAGCGGTCGCTCTACCATCTGAGCTACATCCCC -212
pBS+6/+12 ACCCGGGTGAAAAAGTTGGAGAAGTTGGGTATCGATCCCAATACCTACCGCATGCTAAGCGGTCGCTCTACCATCTGAGCTACATCCCC -212
pBS-128/-138 ACCCGGGTGAAAAAGTTGGAGAAGTTGGGTATCGATCCCAATACCTACCGCATGCTAAGCGGTCGCTCTACCATCTGAGCTACATCCCC -212
pBS-263/-269 ACCCGGGTGAAAAAGTTGGAGAAGTTGGGTACGTCCAACAATACCTACCGCATGCTAAGCGGTCGCTCTACCATCTGAGCTACATCCCC -212
pBS-219/-230 ACCCGGGTGAAAAAGTTGGAGAAGTTGGGTATCGATCCCAATACCTACCGCATGCTAAGCGGTCGCTCTAGGCTATGATATCCATCCCC -212
pBS-150/-156 ACCCGGGTGAAAAAGTTGGAGAAGTTGGGTATCGATCCCAATACCTACCGCATGCTAAGCGGTCGCTCTACCATCTGAGCTACATCCCC -212
pBS-105/-116 ACCCGGGTGAAAAAGTTGGAGAAGTTGGGTATCGATCCCAATACCTACCGCATGCTAAGCGGTCGCTCTACCATCTGAGCTACATCCCC -212
pDBS ACCCGGGTGAAAAAGTTGGAGAAGTTGGGTATCGATCCCAATACCTACCGCATGCTAAGCGGTCGCTCTACCATCTGAGCTACATCCCC -212
Box B
pComp GACGCGGTAACGTCCCAATACACTAGAATGTAGGAAAAGATGCTTTCGACGAGGTTCGAACCCCGAAGGAATCCCGCCCTGGTCTCCAA -123
pBS+6/+12 GACGCGGTAACGTCCCAATACACTAGAATCTAGGAAAAGATGCTTTCGACGAGGTTCGAACCCCGAAGGAATCCCGCCCTGGTCTCCAA -123
pBS-128/-138 GACGCGGTAACGTCCCAATACACTAGAATCTAGGAAAAGATGCTTTCGACGAGGTTCGAACCCCGAAGGAATCGGGCGATCCTATCCAA -123
pBS-263/-269 GACGCGGTAACGTCCCAATACACTAGAATCTAGGAAAAGATGCTTTCGACGAGGTTCGAACCCCGAAGGAATCCCGCCCTGGTCTCCAA -123
pBS-219/-230 GACGCGGTAACGTCCCAATACACTAGAATCTAGGAAAAGATGCTTTCGACGAGGTTCGAACCCCGAAGGAATCCCGCCCTGGTCTCCAA -123
pBS-150/-156 GACGCGGTAACGTCCCAATACACTAGAATCTAGGAAAAGATGCTTTCGACGAGGTCGTCCAACCGAAGGAATCCCGCCCTGGTCTCCAA -123
pBS-105/-116 GACGCGGTAACGTCCCAATACACTAGAATCTAGGAAAAGATGCTTTCGACGAGGTTCGAACCCCGAAGGAATCCCGCCCTGGTCTCCAA -123
pDBS GACGCGGTAACGTCCCAATACACTAGAATCTAGGAAAAGATGCTTTCGACGAGGTCGTCCAACCGAAGGAATCCCGCCCTGGTCTCCAA -123
Box A
tRNA-like
pComp ACGGTACCTGTTGGGCTAAAAAAAAACGCCGCGGTTTCCGCGGTGCGTAAGCGGCACGGCAGTGAGGAAACCGCTGATTATAAGTAGTG -34
pBS+6/+12 ACGGTACCTGTTGGGCTAAAAAAAAACGCCGCGGTTTCCGCGGTGCGTAAGCGGCACGGCAGTGAGGAAACCGCTGATTATAAGTAGTG -34
pBS-128/-138 ACGGTACCTGTTGGGCTAAAAAAAAACGCCGCGGTTTCCGCGGTGCGTAAGCGGCACGGCAGTGAGGAAACCGCTGATTATAAGTAGTG -34
pBS-263/-269 ACGGTACCTGTTGGGCTAAAAAAAAACGCCGCGGTTTCCGCGGTGCGTAAGCGGCACGGCAGTGAGGAAACCGCTGATTATAAGTAGTG -34
pBS-219/-230 ACGGTACCTGTTGGGCTAAAAAAAAACGCCGCGGTTTCCGCGGTGCGTAAGCGGCACGGCAGTGAGGAAACCGCTGATTATAAGTAGTG -34
pBS-150/-156 ACGGTACCTGTTGGGCTAAAAAAAAACGCCGCGGTTTCCGCGGTGCGTAAGCGGCACGGCAGTGAGGAAACCGCTGATTATAAGTAGTG -34
pBS-105/-116 ACGGTAGGCGATGGTATCAAAAAAAACGCCGCGGTTTCCGCGGTGCGTAAGCGGCACGGCAGTGAGGAAACCGCTGATTATAAGTAGTG -34
pDBS ACGGTAGGCGATGGTATCAAAAAAAACGCCGCGGTTTCCGCGGTGCGTAAGCGGCACGGCAGTGAGGAAACCGCTGATTATAAGTAGTG -34
U2 snRNA
pComp CATGTGGTTTCTTTTGTCTGTGGTGGTACTAACATATCTTCTCGGCTATTTAGCTAAGATCATGTTTATAAACTGTTCTTATCAGAGTA +56
pBS+6/+12 CATGTGGTTTCTTTTGTCTGTGGTGGTACTAACATATCCATGGAACTATTTAGCTAAGATCATGTTTATAAACTGTTCTTATCAGAGTA +56
pBS-128/-138 CATGTGGTTTCTTTTGTCTGTGGTGGTACTAACATATCTTCTCGGCTATTTAGCTAAGATCATGTTTATAAACTGTTCTTATCAGAGTA +56
pBS-263/-269 CATGTGGTTTCTTTTGTCTGTGGTGGTACTAACATATCTTCTCGGCTATTTAGCTAAGATCATGTTTATAAACTGTTCTTATCAGAGTA +56
pBS-219/-230 CATGTGGTTTCTTTTGTCTGTGGTGGTACTAACATATCTTCTCGGCTATTTAGCTAAGATCATGTTTATAAACTGTTCTTATCAGAGTA +56
pBS-150/-156 CATGTGGTTTCTTTTGTCTGTGGTGGTACTAACATATCTTCTCGGCTATTTAGCTAAGATCATGTTTATAAACTGTTCTTATCAGAGTA +56
pBS-105/-116 CATGTGGTTTCTTTTGTCTGTGGTGGTACTAACATATCTTCTCGGCTATTTAGCTAAGATCATGTTTATAAACTGTTCTTATCAGAGTA +56
pDBS CATGTGGTTTCTTTTGTCTGTGGTGGTACTAACATATCTTCTCGGCTATTTAGCTAAGATCATGTTTATAAACTGTTCTTATCAGAGTA +56
tag
pComp ACTCCTGATACTGCCTTCGGGCAAAGGTCTGACCTCGACTCTAATAGAAATTACAACTCAAGGTTGTTTCCTTGGAGTTCCACGTTTCC +145
pBS+6/+12 ACTCCTGATACTGCCTTCGGGCAAAGGTCTGACCTCGACTCTAATAGAAATTACAACTCAAGGTTGTTTCCTTGGAGTTCCACGTTTCC +145
pBS-128/-138 ACTCCTGATACTGCCTTCGGGCAAAGGTCTGACCTCGACTCTAATAGAAATTACAACTCAAGGTTGTTTCCTTGGAGTTCCACGTTTCC +145
pBS-263/-269 ACTCCTGATACTGCCTTCGGGCAAAGGTCTGACCTCGACTCTAATAGAAATTACAACTCAAGGTTGTTTCCTTGGAGTTCCACGTTTCC +145
pBS-219/-230 ACTCCTGATACTGCCTTCGGGCAAAGGTCTGACCTCGACTCTAATAGAAATTACAACTCAAGGTTGTTTCCTTGGAGTTCCACGTTTCC +145
pBS-150/-156 ACTCCTGATACTGCCTTCGGGCAAAGGTCTGACCTCGACTCTAATAGAAATTACAACTCAAGGTTGTTTCCTTGGAGTTCCACGTTTCC +145
pBS-105/-116 ACTCCTGATACTGCCTTCGGGCAAAGGTCTGACCTCGACTCTAATAGAAATTACAACTCAAGGTTGTTTCCTTGGAGTTCCACGTTTCC +145
pDBS ACTCCTGATACTGCCTTCGGGCAAAGGTCTGACCTCGACTCTAATAGAAATTACAACTCAAGGTTGTTTCCTTGGAGTTCCACGTTTCC +145
pComp AGGGGAGCAACTCCTTTTTTTCTCTAGAT +173
pBS+6/+12 AGGGGAGCAACTCCTTTTTTTCTCTAGAT +173
pBS-128/-138 AGGGGAGCAACTCCTTTTTTTCTCTAGAT +173
pBS-263/-269 AGGGGAGCAACTCCTTTTTTTCTCTAGAT +173
pBS-219/-230 AGGGGAGCAACTCCTTTTTTTCTCTAGAT +173
pBS-150/-156 AGGGGAGCAACTCCTTTTTTTCTCTAGAT +173
pBS-105/-116 AGGGGAGCAACTCCTTTTTTTCTCTAGAT +173
pDBS AGGGGAGCAACTCCTTTTTTTCTCTAGAT +173
