# Supplementary material for: Transcription of Leishmania major U2 small nuclear RNA gene is directed by extragenic sequences located within a tRNA-like and a tRNA-Ala gene
Source: Parasit Vectors. 2016 Jul 19;9:401. doi: 10.1186/s13071-016-1682-3 (PMC4950102; doi:10.1186/s13071-016-1682-3)
Supplement: Additional file 5: Figure S5. — Sequence comparisons of U2 snRNA genes and flanking regions from T. cruzi (CL Brener Non-Esmeraldo-like). Sequences from the genes located on chromosomes 23, 37 and 6 are shown. The U2 snRNA gene from chromosome 23 is presented in blue font. The position of boxes A and B is indicated. Sequence numbers are relative to the TSS (+1) from the U2 snRNA. (PDF 1404 kb) [file 13071_2016_1682_MOESM5_ESM.pdf]

```
Tcr chr23  CCGTGCAAAATGAATCCCCGCTTTTTTTTAGGCTTCTTTAATGTGGAGTTGTGTTGGTCTGTTTTTTGGGTTTGGGAA.AAAGTTGGCG -179
Tcr chr37  ATGTGCCCGACACGCCCTAAACACGTT...GCTTTCGTTTTCCAGGGACAACGCAAATGCGCTCGATTGTTCGAGGAGCAAAAGTAACA -164
Tcr chr6   ATGTGCCCGACACGCCCTAAACATGCC...GCTTTCGTTTTCCAGAAGACAACGCAAATGCGCCCGATTGTTCGAGGAGCAAAAGCAACA -162
Conserved    gtgc    a     cc              g  ttc tt     g         g     t  g      t g t   gga  aaa     c

                  Box B                                                      Box A
Tcr chr23  TTTCTTTGGAATCGAACCCGTGACCCTTGGCAATGTTTGTTGCGGACTCTGGAGCGTCTTAGACATTTAAGCTAAGAAGTAACGTTGCCC -89
Tcr chr37  CTTCGTAAGCTATAAACACATAT.........GTATCCAATCCGAA...TAGAGCGT..TGAACAATTAAGCGAGGAAGGGACGGAAGAA -88
Tcr chr6   CTTTGTAAGCTGTAAATACTT...........GTATCCAATCCGAA...TAGAGCGT..TGAACAATTAAGCGAGGAGGGGACGGACGAA -88
Conserved   tt  t  g      aa  c t              t t    t cg a   t gagcgt  t  aca ttaagc a ga g  acg

                                                                                      U2 snRNA →
Tcr chr23  GTCGTTTGTAAGATTACGATGGAAAAATTCCCCATTTGGTTTCGAAACTATTTTTACGAGGTTACACTGTTATTGGAGACGGCTGTGTAT +2
Tcr chr37  TTCTTTAGCAAGATTGCGATC.AAAAATTCCCCATTTGGTTTCGAAACTATTTTTACGAGGTTACTTTTTTATTGGAGCTGTTGTGTGT +2
Tcr chr6   TTCTTTAGCAAGATTGCGATC.AAAAACTCCCCATTTGGTTTTGAAACTATTTTTACGAGGTTACTTTTTTATTGGGAGCTGTTGTGTGT +2
Conserved   tc tt g aagatt cgat  aaaaa tccccatttggttt gaaactatttttacgaggttac  t ttattgg   c g tgtgt t

Tcr chr23  ATCTTCTCGGCTATTTAGCTAAGATCAAGTTTTTAAACTGTTCTTATCAGAGTAATTCCTGATATGGGCTTCGTCCCAGGAATAGAAACC +92
Tcr chr37  ATCTTCTCGACTATTTAGCTAAGATCAAGTTTTTAAACTGTTCTTATCAGAGTAATTCCTGATATGGGCTTCGTCCCAGGAATAGAAACC +92
Tcr chr6   ATCTTCTCGACTATTTAGCTAAGATCAAGTTTTTAAACTGTTCTTATAAGAGTAATTCCTGACATGGGCTTCATCCCAGGAATAAAAACC +92
Conserved  atcttctcg ctatttagctaagatcaagtttttaaactgttcttat agagtaattcctga atgggcttc tcccaggaata aaacc

Tcr chr23  GCTGATCTCTCAAGCGTTCTTCCGGAGTTCCACGTGTCCGGATGGAGCGCTTCCTTTTCCAAAATCGTGTTCGCTTTTACAAAAT..... +177
Tcr chr37  GCTGATCTCTCAGGCGTTCTTCCGGAGTTTCGCGTGTCCGGATGGAGCGTCTCCTTTTCCAAAATTGTGTTCGCTTTTTCCTTTTTTTTT +182
Tcr chr6   GCTGATCTCTCAGGCGTTCTTCCGGAGTTTCGCGCGTCCGGATGGAGCGTCTCCT...CCAAAATTGTGTTCGCTTTTTCCTTTTTTTC. +178
Conserved  gctgatctctca gcgttcttccggagtt c cg gtccggatggagcg  tcct   ccaaaat gtgttcgctttt c    t
```
