# Supplementary material for: Transcription of Leishmania major U2 small nuclear RNA gene is directed by extragenic sequences located within a tRNA-like and a tRNA-Ala gene
Source: Parasit Vectors. 2016 Jul 19;9:401. doi: 10.1186/s13071-016-1682-3 (PMC4950102; doi:10.1186/s13071-016-1682-3)
Supplement: Additional file 1: Figure S1. — Sequence alignment of U2 snRNA genes from trypanosomatids. Sequences were obtained from the TriTrypDB databases of the following species of trypanosomatids: L. major Friedlin (Lmj), L. arabica LEM1108 (Lar), L. tarentolae Parrot-TarII (Ltr), L. infantum JPCM5 (Lin), L. amazonensis MHOMBR/71973/M2269 (Laz), L. donovani BPK282A1 (Ldn), L. aethiopica L147 (Lae), L. mexicana U1103 (Lmx), L. panamensis MHOM/COL/81/L13 (Lpn), L. braziliensis M2904 (Lbr), L. enriettii LEM3045 (Len), E. monterogeii LV88 (Emg), C. fasciculata Cf-Cl (Cfs), L. pyrrhocoris H10 (Lpy), L. seymouri ATCC 30220 (Lsy), L. collosoma (unspecified strain) [39] (Lcl), T. cruzi CL Brener Non-Esmeraldo-like, copy of the chromosome 23 (Tcr), T. vivax Y486 (Tvx), T. congolense IL3000 (Tcn), T. evansi STIB 805 (Tev) and T. brucei DAL972 (Tbr). The alignment was obtained with the DNAMAN software, and manually corrected. Arrows indicate the identified pseudouridines (Ψ) in the L. major U2 snRNA (this work). BPRR and Sm sites are also indicated. The 3' ends (C residues) that have been experimentally mapped are shown in red font for L. major (this work), T. cruzi [45] and T. brucei [44]. The T-tract length is indicated for each species. Sequence numbers are relative to the TSS (+1) from the U2 snRNA. This alignment was used to generate the sequence logo depicted in Fig. 3b. (PDF 2137 kb) [file 13071_2016_1682_MOESM1_ESM.pdf]

```
                     Ψ14                 Ψ34 Ψ36                  Ψ55                                       Ψ86     Ψ93
Lmj  ATATCTTCTCGGCTATTTAGCTAAGATCATGTTTAT.AAACTGTTCTTATCAGAGTAACTCCTGATACT.GCCTTCGGGCAA..AGGAATA.GAAATTAC.AACTC.AA  102
Lar  ATATCTTCTCGGCTATTTAGCTAAGATCATGTTTAT.AAACTGTTCTTATCAGAGTAACTCCTGATACT.GCCTTCGGGCAA..AGGAATA.GAAATTAC.AACCC.AA  102
Ltr  ATATCTTCTCGGCTATTTAGCTAAGATCATGTTTAT.AAACTGTTCTTATCAGAGTAACTCCTGATACT.GCCTTCGGGCAA..AGGAATA.GAAATTAT.AACTC.AA  102
Lin  ATATCTTCTCGGCTATTTAGCTAAGATCATGTTTAT.AAACTGTTCTTATCAGAGTAACTCCTGATACT.GCCTTCGGGCAA..AGGAATA.GAAATTAT.AACCC.AA  102
Laz  ATATCTTCTCGGCTATTTAGCTAAGATCATGTTTAT.AAACTGTTCTTATCAGAGTAACTCCTGATACT.GCCTTCGGGCAA..AGGAATA.GAAATTAT.ATCTC.AA  102
Ldn  ATATCTTCTCGGCTTTTTAGCTAAGATCATGTTTAT.AAACTGTTCTTATCAGAGTAACTCCTGATACT.GCCTTCGGGCAA..AGGAATA.GAAATTAT.AACCC.AA  102
Lae  ATATCTTCTCGGCTATTTAGCTAAGATCATGTTTAT.AAACTGTTCTTATCAGAGTAACTCCTGATACT.GCCTTCGGGCAA..AGGAATA.GAAATTAT.AATCC.AA  102
Lmx  ATATCTTCTCGGCTATTTAGCTAAGATCATGTTTAT.AAACTGTTCTTATCAGAGTAACTCCTGATACT.GCCTTCGGGCAA..AGGAATA.GAAATTAT.ATCTC.AA  102
Lpn  ATATCTTCTCGGCTATTTAGCTAAGATCATGTTTAT.AAACTGTTCTTATCAGAGTAACTCCTGATACT.GCCTTCGGGCAA..AGGAATA.GAAATTAT.AAGTC.AA  102
Lbr  ATATCTTCTCGGCTATTTAGCTAAGATCATGTTTAT.AAACTGTTCTTATCAGAGTAACTCCTGATACT.GCCTTCGGGCAA..AGGAATA.GAAATTAT.AAGTC.GA  102
Len  ATATCTTCTCGGCTTTTTAGCTAAGATCATGTTTAT.AAACTGTTCTTATCAGAGTAACTCCTGATACT.GCCTTCGGGCAA..AGGAATA.GAAATTAT.GAGCC.AA  102
Emg  ATATCTTCTCGGCTATTTAGCTAAGATCATGTTTAT.AAACTGTTCTTATCAGAGTAACTCCTGATACT.GCCTTCGGGCAA..AGGAATT.GAAATTAT.AAGCCCAA  103
Cfs  ATATCTTCTCGGCTTTTTAGCTAAGATCATGTTTAT.AAACTGTTCTTATCAGAGTAACTCCTGATATT.GCCTTCGGGCAA..AGGAATA.CAAATGAT.CGCAC.TA  102
Lpy  ATATCTTCTCGGCTATTTAGCTAAGATCATGTTTAT.AAACTGTTCTTATCAGAGTAACTCCTGATATT.GCCTTCGGGCAA..AGGAATA.GAAATCAT.GCACC.TA  102
Lsy  ATATCTTCTCGGCTATTTAGCTAAGATCATGTTTAT.AAACTGTTCTTATCAGAGTAACTCCTGATATT.GCCTTCGGGCAA..AGGAATA.GAAATCAT.GGTAC.TA  102
Lcl  ATATCTTCTCGGCTTTTTAGCTAAGATCATGT.TTTTAAATGTTCTTATCAGAGTAACTCCTGATATTTGCCTTCGGGCAATTAGGAATACGAAATCTTTGAT.C.AC  106
Tcr  ATATCTTCTCGGCTATTTAGCTAAGATCAAGT.TTTTAAACTGTTCTTATCAGAGTAATTCCTGATATGGGCTTCGT..CCC..AGGAATA.GAAACCGCTGATCT.CT  102
Tvx  ATATCTTCTCGGCTATTTAGCTAAGATCAAGT.TATTAAACTGTTCTTATCAGAGTAACTCCTGATACGGGCGTTCGCGCCC..AAGGATA.GTAACTTT.GATGCGAG  104
Tcn  ATATCTTCTCGGCTATTTAGCTAAGATCAAGT.TATTAAACTGTTCTTATCAGAGTAACTCCTGATATGGGCCTCGG..CCC..AAGGATA.GTAACTGTTGAGGC.GC  102
Tev  ATATCTTCTCGGCTATTTAGCTAAGATCAAGT.TATTAAACTGTTCTTATCAGAGTAACTCCTGATACGGGCCTTTG.GCCC..AAGGATC.TAAACTGTTGGCTG.TC  103
Tbr  ATATCTTCTCGGCTATTTAGCTAAGATCAAGT.TATTAAACTGTTCTTATCAGAGTAACTCCTGATACGGGCCTTTG.GCCC..AAGGATC.TAAACTGTTGGCTG.TC  103
     atatcttctcggct tttagctaagatca gt t t aaa tgttcttatcagagtaa tcctgata   gc t     c    a g at     aa
                                     BPRR                                                              Sm
```

```
                                                                                              T tract length
Lmj  GG.TTGTTTCCTTGGAGTTCCAC.GTTTCCAGGGGAGCAACTCC...............TTTTTTCGCCCAG  157    Lmj  T6
Lar  GG.TTGTTTCCCTGGAGTTCCAC.GTTTCCAGGGGAGCAACTCC...............TTTTTTAGCCCAG  157    Lar  T6
Ltr  GG.TTGTTTCCCTGGAGTTCCAC.GTTTCCAGGGGAGCAACTC................TTTTTTTTTCATT  156    Ltr  T9
Lin  GG.TTGTTTCCCTGGAGTTCCAC.GTTTCCAGGGGAGCAACTC................TTTTTTTCGCCCG  156    Lin  T7
Laz  GG.TTGTTTCCCTGGAGTTCCAC.GTTTCCAGGGGAGCAACTC................TTTTTTCCTCCTA  156    Laz  T6
Ldn  GG.TTGTTTCCCTGGAGTTCCAC.GTTTCCAGGGGAGCAACTCC...............TTTTTTCGCCCAG  157    Ldn  T6
Lae  GG.TTGTTTCCCTGGAGTTCCAC.GTTTCCAGGGGAGCAACTCC...............TTTTTTCGCCCAG  157    Lae  T6
Lmx  GG.TTGTTTCCCTGGAGTTCCAC.GTGTCCAGGGGAGCAACTC................TTTTTTTCTCCCA  156    Lmx  T7
Lpn  GG.TTGTTTCCCTGGAGCTCCAC.CTTTCCAGGGGAGCAACTTC...............TTTTTTTTTCCCG  157    Lpn  T9
Lbr  GG.TTGTTTCCCTGGAGCTCCAC.CTTTCCAGGGGAGCAACTTC...............TTTTTTTTCCCGG  157    Lbr  T8
Len  GG.TTGTTTCCCTGGAGTTCCAC.CTTTCCAGGGGAGCAACTTTAC.............TTTTTACCCCACC  159    Len  T5
Emg  GG.TTGCTTCCCTGGAGTTCCAC.CGTTCCAGGGGAACAACTTCAC.............TTTTTTTTTTTTC  160    Emg  T12
Cfs  GG.TTGTTTCCCTGGAGTTCCAC.CTTTCCAGGGGAGCAACTGCACC............TTTTTAGATTTTG  160    Cfs  T5
Lpy  GG.TTGTTTCCCTGGAGTTCCAC.CTTTCCAGGGGAGCAACTGCC..............TTTTTTGCATTCC  158    Lpy  T6
Lsy  GG.TTGTTTCTCTGGAGTTCCAC.CTTTCCAGGGGAGCAGCTACC..............TTTTCGCACAGAC  158    Lsy  T4
Lcl  GC.GAGTTTTCCTGGAGTTCCACTCTTTCCAGGCGAAGCTCGCCC..............TTTTTTTTTCTTT  163    Lcl  T9
Tcr  CAAGCGTTCTTCCGGAGTTCCAC.GTGTCCGGATGGAGCGC.TTCC.............TTTTCCAAAATCG  159    Tcr  T4
Tvx  CC.GCGTTCTTCCGGAGTTCCAC.GTGTCCGGACTGAGCGTTCGTAGGCTCCAAAAACGTTTTTGAATAATC  174    Tvx  T5
Tcn  CG.GCGTCCTCCCGGAGTTCCAC.GTGTCCGGTTGGGGCG....CCGCCTCCAATCG..TTTTCCGCCGCTC  166    Tcn  T4
Tev  CC.GCGTTCTTCCGGGGTTCCAC.TTGTCCGGATGGAGCG..CGACGGTCGCGAACA..TTTTCCGACAAAA  169    Tev  T4
Tbr  CC.GCGTTCTTCCGGGGTTCCAC.TTGTCCGGATGGAGCG..CGACGGTCGCGAACA..TTTTCCGACAAAA  169    Tbr  T4
         g         gg g tccac     tcc g                      tttt
```
